# PEGylated AdipoRon derivatives improve glucose and lipid metabolism under insulinopenic and high-fat diet conditions

**DOI:** 10.1016/j.jlr.2021.100095

**Published:** 2021-06-30

**Authors:** Toshiharu Onodera, Ebrahim Ghazvini Zadeh, Peng Xu, Ruth Gordillo, Zheng Guo, Nolwenn Joffin, Biao Yu, Philipp E. Scherer, Wen-hong Li

**Affiliations:** 1 Touchstone Diabetes Center, Department of Internal Medicine, The University of Texas Southwestern Medical Center, Dallas, TX, USA; 2 Department of Cell Biology, The University of Texas Southwestern Medical Center, Dallas, TX, USA; 3 State Key Laboratory of Bio-organic and Natural Products Chemistry, Shanghai Institute of Organic Chemistry, Chinese Academy of Sciences, Shanghai, China; 4 Department of Biochemistry, The University of Texas Southwestern Medical Center, Dallas, TX, USA

**Keywords:** diabetes, lipid metabolism, ceramides, adiponectin, drug therapy/hypolipidemic drugs, insulin resistance, high-fat diet, drug optimization, INS-1 beta cells, sphingosine-1-phosphate, AdipoR1/2, adiponectin receptor1/2, AMPK, AMP-activated protein kinase, BAT, brown adipose tissue, BSA, bovine serum albumin, HFD, high-fat diet, ITT, insulin tolerance test, OGTT, oral glucose tolerance test, PA, palmitic acid, PEG, polyethyleneglycol, PI, propidium iodide, PPAR, peroxisome proliferator-activated receptor, scWAT, subcutaneous white adipose tissue, STZ, streptozotocin, SVF, stromal vascular fraction, TGTT, triglyceride tolerance test

## Abstract

The pleiotropic actions of adiponectin in improving cell survival and metabolism have motivated the development of small-molecule therapeutic agents for treating diabetes and lipotoxicity. AdipoRon is a synthetic agonist of the adiponectin receptors, yet is limited by its poor solubility and bioavailability. In this work, we expand on the protective effects of AdipoRon in pancreatic β-cells and examine how structural modifications could affect the activity, pharmacokinetics, and bioavailability of this small molecule. We describe a series of AdipoRon analogs containing amphiphilic ethylene glycol (PEG) chains. Among these, AdipoRonPEG5 induced pleiotropic effects in mice under insulinopenic and high-fat diet (HFD) conditions. While both AdipoRon and AdipoRonPEG5 substantially attenuate palmitate-induced lipotoxicity in INS-1 cells, only AdipoRonPEG5 treatment is accompanied by a significant reduction in cytotoxic ceramides. *In vivo*, AdipoRonPEG5 can substantially reduce pancreatic, hepatic, and serum ceramide species, with a concomitant increase in the corresponding sphingoid bases and improves insulin sensitivity of mice under HFD feeding conditions. Furthermore, hyperglycemia in streptozotocin (STZ)-induced insulinopenic adiponectin-null mice is also attenuated upon AdipoRonPEG5 treatment. Our results suggest that AdipoRonPEG5 is more effective in reducing ceramides and dihydroceramides in the liver of HFD-fed mice than AdipoRon, consistent with its potent activity in activating ceramidase in vitro in INS-1 cells. Additionally, these results indicate that the beneficial effects of AdipoRonPEG5 can be partially attributed to improved pharmacokinetics as compared with AdipoRon, thus suggesting that further derivatization may improve affinity and tissue-specific targeting.

## Graphical abstract

**Figure:**
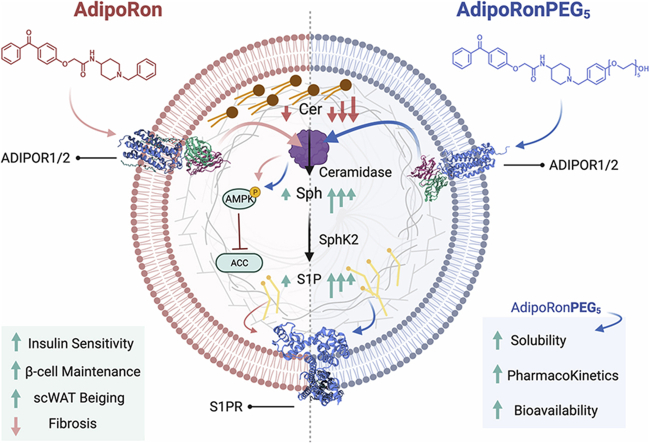

Sedentary lifestyle (1) and high-fat diet (HFD) (2, 3) result in increased adiposity, associated with insulin resistance, hyperglycemia, and an increased risk of type 2 diabetes. The pathogenesis of this disease is further augmented by glucolipotoxicity-induced apoptosis of pancreatic β-cells and inadequate production or secretion of insulin (4, 5). The adipocyte-derived protein hormone, adiponectin, counteracts fatty acid and cytokine-induced apoptosis in the pancreatic β-cell line INS-1 (6), and protects MIN6 cells and mouse islets from serum-starvation-induced apoptosis (7). Adiponectin levels are reduced as obesity progresses and adipocytes become dysfunctional (5, 8). Interestingly, adiponectin was shown to maintain β-cell mass and glucose homeostasis in *ob*/*ob* mice when a 2–3 fold overexpression of adiponectin was induced (9). Adiponectin is therefore regarded as a promising antidiabetic adipokine.

Adiponectin receptor1 (AdipoR1) and adiponectin receptor2 (AdipoR2) are the main receptors of adiponectin through which molecular and cellular actions take place, with downstream effectors including ceramidase and sphingolipids, AMP-activated protein kinase (AMPK), *peroxisome proliferator-activated receptor* (*PPAR*) alpha (*PPAR*α), *PPAR gamma* (*PPARγ*), and additional components (10, 11). Overexpression and siRNA knockdown of *AdipoR1* and *AdipoR2* revealed their important roles in cellular binding of adiponectin, as well as the downstream signaling events involving AMPK and *PPAR*α. Many studies point to metabolically beneficial effects of both AdipoR1/2. We previously defined the very potent ceramide lowering effects induced by adiponectin signaling through its receptors (12). These effects on sphingolipid concentrations can explain most physiological and cell signaling effects, such as the insulin sensitizing, anti-inflammatory, and antiapoptotic actions attributed to adiponectin. Evidence is emerging for the pleiotropic roles of ceramides and the other sphingolipids in the development of insulin resistance and other metabolic disorders (13). More recent data showed that adiponectin was essential to maintain minimal lipid homeostasis under insulinopenic conditions to improve local lipid metabolism in the islets (14) and the process acts as an important antilipotoxic phenomenon to boost β-cell regeneration primarily mediated by adiponectin's action, at least in part, on the β-cells directly (15). This is consistent with the direct action of adiponectin on β-cells as both *AdipoR1* and *AdipoR2* are abundantly expressed in islet β-cells (16, 17).

In addition to its pleiotropic protective effects on obesity-related metabolic disorders, adiponectin elicits a central role in the cold-induced browning of subcutaneous white adipose tissue (scWAT), contributing to reduced obesity in mice (18, 19). Upon chronic cold exposure, adiponectin is sequestered toward the stromal vascular fraction (SVF) of scWAT, where adiponectin is predominately tethered to M2 macrophages in a T-cadherin/Akt-dependent manner. This results in the proliferation and accumulation of M2 macrophages in the scWAT. This accumulation is accompanied by upregulated expression of tyrosine hydroxylase for production of catecholamine, a key factor required for browning of WAT (18). However, a more recent study indicated that M2 macrophages do not synthesize relevant amounts of catecholamines, deeming them unlikely to have a direct role in adaptive thermogenesis (20). Regardless of whether M2 macrophages are relevant local thermogenic efferent during cold-induced browning in scWAT, hypoadiponectinemia was shown to result in a significant reduction in energy expenditure (21).

Various factors prevent the use of adiponectin as an attractive treatment for obesity-related disorders. Injection of recombinant adiponectin, though therapeutically effective, is not attractive due to its relatively high plasma concentrations, short half-life, and its complex quaternary structure, which requires proper posttranslational modifications for multimerization (22). Interestingly, recent efforts by Kadowaki and colleagues have identified an orally active low-molecular-weight compound known as AdipoRon that activates *AdipoR1* and *AdipoR2* as well as the downstream *AMPK* and *PPARγ* pathways in the liver and skeletal muscles (23). Several studies have investigated the metabolic actions of AdipoRon as well as the associated protective roles against liver inflammation and fibrosis (24, 25) diabetic nephropathy and kidney disease (26, 27) atherosclerosis, myocardial ischemia/reperfusion (28) cardiac hypertrophy (29) spinal cord injury (30) corticosterone-induced depression (31) cognitive dysfunction of Alzheimer's disease (32) and bone (33) ovarian (34) and pancreatic cancer (35).

Here, we explored whether AdipoRon, upon exposure to fatty acids, can act as an exogenous modulator of β-cell responses by activating AdipoR-elicited cellular ceramidase activity (12, 36). Additionally, we employed a cell-based assay to determine how structural modifications may generate more potent AdipoRon derivatives that can thwart the toxic effect of palmitate on the pancreatic β-cell line INS-1. This led us to investigate the favorable effects of an AdipoRon derivative, namely AdipoRonPEG_5_, on glucose and lipid homeostasis in INS-1 cells, STZ-induced insulinopenic adiponectin knockout mice, and HFD-induced obese mouse models. In addition, we examined the hepatic AdipoR1/AdipoR2 expression and the downstream pathway activation in type 2 diabetic mice. The data suggests that AdipoRonPEG_5_ ameliorates gluco- and lipotoxicity by activating the AMPK pathway through AdipoR1 and by upregulating ceramidase expression.

## Materials and methods

### Materials

Palmitic acid (PA) was supplied by Sigma-Aldrich (P5585). The recombinant C-terminal globular domain of adiponectin (gAcrp30) was a product from Novus Biologicals (NBP199295). Bovine serum albumin (BSA, fatty-acid free) was obtained from Sigma-Aldrich (A8806). Propidium iodide (PI) for discrimination of necrotic from apoptotic cells was product of Invitrogen (P3566). RPMI Phenol Red-free 1640 medium and penicillin/streptomycin were provided by Gibco (11835055) and Invitrogen (15140163), respectively. AdipoRon and its analogues including AdipoRonPEG_5_ were synthesized as described in the supplemental data.

### Mice

All mice including the adiponectin knockout mouse (37) were bred in the C57BL/6 genetic background. Mice were fed on regular (LabDiet #5058) or high fat (60%, Research #D12492). HFD was started from 6 weeks. Mice were maintained in 12-h dark/light cycles, with ad libitum access to diet and water. All protocols for mouse use and euthanasia were reviewed and approved by the Institutional Animal Care and Use Committee.

### logP prediction and solubility measurements of AdipoRon and AdipoRonPEG_5_

We computed octanol-water partition coefficients (logP) using Molinspiration, which was developed by fitting calculated logP with experimental logP for a training set of more than 12,000, mostly drug-like molecules (https://www.molinspiration.com). We quantified the solubilities of AdiponRons by LC-MS/MS. Briefly, water (HPLC grade) was added in 50–100 μl increments to a glass vial containing either AdipoRon (0.97 mg) or AdipoRonPEG_5_ (1.69 mg), vortexed until the compound started to dissolve (AdipoRon, 3,100 ml; AdipoRonPEG_5_, 400 ml). The suspension was then stirred overnight away from light. The solution was transferred to Teflon tubes and centrifuged at 15,000 rpm at room temperature for 10 min. We then collected the supernatant (aqueous saturated solution) for dilution and analysis.

For standards, 98 μl water was added to an Eppendorf tube and combined with 200 μl of methanol containing 0.15% formic acid and 75 ng/ml Initial Standard (IS, n-benzeylbenzamide in MeOH. IS final conc. = 50 ng/ml). Samples were spiked with 2 μl of IS. The samples were vortexed 15 s, and spun 2 × 13,200 rpm in a standard microcentrifuge. The supernatant was then analyzed by LC-MS/MS on a QTrap 4000 system (AB Sciex) with the following settings: Ion Source/Gas Parameters: CUR = 25, CAD = medium, IS = 5,000, TEM = 500, GS1 = 70, GS2 = 70. Buffer A: Water + 0.1% formic acid + 2 mM NH_4_OAC; Buffer B: methanol + 0.1% formic acid + 2 mM NH_4_OAC; flow rate 1.5 ml/min; Agilent C18 XDB column, 5 micron packing 50 × 4.6 mm size. HPLC elution gradient: 0–0.5 min 97% A, 0.5–2.0 min gradient to 100% B, 2.0–3.5 min 100% B, 3.5–3.6 min gradient to 97% A, 3.6–4.1 97% A. Compound transition 429.156 to 174.1 for AdipoRon and 665.289 to 135.2 for AdipoRonPEG_5_.

### Pharmacokinetics and liver bioavailability

Two groups of male C57BL/6J mice (21 mice/group) at 8 weeks of age were dosed IP with 20 mg/kg AdipoRon or AdipoRonPEG_5_, 0.2 ml/mouse formulated as 10% DMSO/10% Cremophor/20% PEG400/60% PBS. Whole blood was harvested at seven time points post IP dosing, 10 min, 30 min, 90 min, 180 min, 360 min, 960 min, and 1,440 min. Three mice were euthanized at each time point. Plasma was processed from whole blood by centrifugation of the ACD-treated blood for 10′ at 10,000 rpm in a standard centrifuge. Additionally, the liver was harvested. The tissues were weighed and snap frozen in liquid nitrogen.

To quantify plasma drug concentration and PK, we prepared standards by adding 98 μl blank plasma (BioIVT) to an eppendorf. These were spiked with 2 μl of initial standard. For QCs, we added 98.8 μl blank plasma to Eppendorf, spiked with 1.2 μl of initial standard. For samples, we mixed 100 μl plasma with 200 μl of methanol containing 0.15% formic acid and 75 ng/ml IS (IS final conc. = 50 ng/ml). The samples were vortexed 15 s, incubated at room temp for 10′, and spun 2× 13,200 rpm in a standard microcentrifuge. The supernatant was then analyzed by LC-MS/MS.

To measure liver drug concentration and bioavailability, we prepared standards by adding 98 μl blank liver homogenate to an Eppendorf, spiked with 2 μl of initial standard. For QCs, we added 98.8 μl blank liver homogenate to an Eppendorf, spiked with 1.2 μl of initial standard. For samples, we mixed 100 μl liver homogenate with 200 μl of methanol containing 0.15% formic acid and 75 ng/ml IS (IS final conc. = 50 ng/ml). The samples were vortexed 15 s, incubated at room temp for 10′, and spun 2× 13,200 rpm in a standard microcentrifuge. The supernatant was then analyzed by LC-MS/MS.

### Genotyping PCR

Approximately 3 mm of mouse tail tip was incubated in 80 μl 50 mM NaOH at 95°C for 1.5 h. In total, 8 μl 1 M Tris-HCl (pH 8.0) was added for neutralization. After vortexing and a short spin down, 0.5–1 μl of supernatant was used as PCR template. Primer sequences for genotyping PCR are listed in supplemental Table S1. The PCR program was: 95°C for 5 min, followed by 35 cycles of 95°C for 15 s, 62°C for 30 s, and 72°C for 30 s, and ended with 72°C for 3 min.

### qPCR

Total RNA was extracted with the RNeasy Mini kit (Qiagen #74106) for the pancreas or Trizol (Invitrogen #15596018) for the liver and adipose tissue. cDNA was synthesized with iScript cDNA Synthesis Kit (Bio-Rad #170-8891). Quantitative real-time PCR (qPCR) was performed with the Powerup SYBR Green PCR master mix (Applied Biosystems # A25742) on Quantistudio 6 Flex Real-Time PCR System (Applied Biosystems *#*4485694). Primer sequences for qPCR are listed in supplemental Table S2.

### Cell culture

The rat insulinoma cell line INS-1 (passages 20–30) was grown in monolayer culture in RPMI 1640 medium supplemented with 10% heat-inactivated fetal bovine serum (FBS), 500 U/ml penicillin, 50 μg/ml streptomycin, 2 mM glutamine, and 50 μM 2-mercaptoethanol in 100 mm petri dishes in an atmosphere of 5% CO_2_ at 37°C. Subconfluent cells were maintained in continuous passages by trypsinization of cultures 7 days after plating. The medium was changed every 42 h. Cell number was determined after cell dissociation with trypsin/EDTA at 37°C. INS-1 cells were routinely seeded at 60× 10^3^ cells/well of a 96-well plate for cell death detection experiments at 60%–70% confluence.

### In vitro β-cell stress by PA and protection with AdipoRons

#### PA/BSA complex solution

A 7.1 mM palmitic acid stock solution was prepared in 95% ethanol by incubating at 37°C. In parallel, 2% fatty-acid-free BSA was solubilized in sterile nanopure water at 37°C. The palmitic acid/BSA complex solution was then prepared by incubating the components in a molar ratio of 5:1 at 37°C in a shaking water bath for 1 h and was used directly for INS-1 stimulation experiments. Control cultures were carried out in the presence of BSA/ethanol in RPMI without palmitic acid.

#### Preparation of INS-1 cells for sphingolipid measurement upon treatment with AdipoRon analogs

INS-1 cells were seeded at 2 × 10^6^/well in a 6-well plate. Cells were treated as follows for 24 h: (i) Control: No FBS or PA (0.25 mM); (ii) Positive Control: 10% FBS + PA (0.25 mM); (iii) Adiponectin (10 nM) + PA (0.25 mM); (iv) AdipoRonPEG_6_HTL (10 nM) + PA (0.25 mM); (v) AdipoRonPEG_6_HTL (1 μM) + PA (0.25 mM); (vi) AdipoRonPEG_5_ (1 μM) + PA (0.25 mM); (vii) AdipoRonPEG_5_ (20 μM) + PA (0.25 mM). Thereafter, treatment was aspirated, and the cells were washed with cold PBS x 2, scraped off with 1–2 ml PBS, and spun down at 2,500 rpm for 5 min. The supernatant was aspirated, and the pallet was stored at 80°C for sphingolipid analysis.

### Quantification of β-cell death by Hoechst 33342/PI staining

Following a 2 h preincubation under serum-free conditions, INS-1 cells were kept for 24 h in Phenol Red-free FBS-free RPMI 0.5% BSA as a control or containing 0.25 mM PA-BSA in the presence or absence of treatment. Treatment includes 10% FBS, 20 nM adiponectin, 10 μM AdipoRon, 10 μM AdipoRonPEG_5_, or 1.0 μM AdipoRonPEG_6_HTL. Cells were double stained for 10 min at 4°C with Hoechst 33342 and propidium iodide (PI) at 10 μg/ml and 1 μg/ml, respectively. Adding Hoechst 33342/PI to Phenol Red-free cell media ensures that any floating dead cells were not lost since aspirating the treatment media is no longer necessary. Images were acquired on a confocal laser scanning microscope (Zeiss LSM 510) in the green (Ex 488 nm, Em 500–530 nm) or red channel (Ex 543 nm, Em 565–615 nm) with a ×40/1.3 objective lens connected to a confocal scanner and a workstation. Hoechst 33342/PI double staining enables the differentiation of apoptotic cells from necrotic cells. Hoechst 33342 stains the condensed chromatin in apoptotic cells more brightly than the chromatin in normal cells. PI is a red-fluorescence dye that is only permeant to dead cells.

Quantification of β-cell death by PI staining was carried out on a fluorescence plate reader. Following a 2 h preincubation under serum-free conditions, INS-1 cells (60k cells/well, 96-well plate (COSTAR ref # 3603)) were kept for 24 h in Phenol Red-free FBS-free RPMI 0.5% BSA as a control or containing 0.25 mM PA-BSA in the presence or absence of treatment. Cells were stained for 10 min at 4°C with propidium iodide (PI) at 1 μg/ml (added to the existing media to avoid loss of dead cells). PI fluorescence intensity was measured with a plate reader (PHERAstar, BMG Labtech).

### Systemic tests

For oral glucose tolerance test (OGTT), mice were fasted for 4–6 h and subjected to an oral gavage of dextrose (2 mg/g body weight). Tail blood was collected at 0, 15, 30, 60, and 120 min and prepared for serum and assayed for glucose and insulin. For insulin tolerance test (ITT), insulin (0.75 U/kg Humulin R; Eli Lilly, Indianapolis, IN) was administered under fed condition. Serum glucose level was measured at 0, 15, 30, 60, and 90 min time point. Triglyceride tolerance test (TGTT) was initiated by oral gavage of 20% Intralipid (10 μl/g BDW, l141-100 ml, Sigma), and serum was collected at 0, 1, 1.5, 3, and 6 h for triglyceride assay. Glucose, insulin, and triacylglyceride levels were measured using an oxidase-peroxidase assay (Sigma P7119), insulin ELISA (Crystal Chem, Elk Grove village, IL, #15596018), and Infinity Triglycerides Reagent (Thermo Fisher Scientific TR22421).

### Western blot

Protein was extracted from the adipose tissue by homogenization in PBS supplemented with 1 mM EDTA, 20 mM NaF, 2 mM Na_3_VO_4_, and protease inhibitor cocktail. 5× RIPA buffer was added to the homogenate for a final concentration of 10 mM Tris-HCl, 2 mM EDTA, 0.3% NP40, 0.3% deoxycholate, 0.1% SDS, and 140 mM NaCl, pH 7.4. The sample was centrifuged at 10,000 *g* for 5 min. In total, 20–50 μg/lane of supernatant protein was separated by SDS-PAGE (NP0335BOX, Thermo Fisher) and transferred to nitrocellulose membrane. The blots were then incubated overnight at 4°C with primary antibodies (1:1,000) in a 1% BSA TBST-blocking solution. Primary antibodies are listed in supplemental Table S3. Primary antibodies were detected using secondary antibodies labeled with infrared dyes emitting at 700 nm or 800 nm (1:5,000, Li-Cor Bioscience 925-68073 and 926-32213, respectively) The Odyssey Infrared Imager was used to visualized western blots with Li-Cor IRdye secondary antibodies.

### STZ treatment

Streptozotocin (STZ; Sigma S1030) was dissolved by 0.05 M citrate buffer (final; 20 mg/ml). Following 6 h fasting, mice received a single intraperitoneal injection of streptozotocin (200 mg/kg) to induce insulinopenic conditions.

### AdipoRon derivative treatment

Six-week-old wild-type male mice were fed HFD for 4 weeks or 7 months In total, 5 mg/Kg of PBS, Adiporon, AdipoRonPEG_5_, AdipoRonPEG_6_HTL were injected twice a day for 5 days. After 5 days of treatment, systemic test is performed, and serum (Before and after treatment), subcutaneous, epididymal, liver, heart samples were harvested for qPCR and sphingolipid analysis.

### Cold exposure

In total, 5 mg/Kg of PBS and AdipoRonPEG_5_ were injected twice a day for 5 days at room temperature followed by housing in the cold chamber (4°C) for 7 days. Body temperature was measured at 0, 1, 2, 3, 4, and 5 h time point at the first day of cold exposure. In total, 5 mg/Kg of PBS and AdipoRonPEG_5_ were injected once a day for 7 days after cold exposure followed by harvesting tissues for qPCR analysis.

### RNA-seq

RNA-sequence was performed by Novogene (Sacramento, CA) by utilizing isolated RNA. After the QC procedures, mRNA from eukaryotic organisms is enriched from total RNA using oligo(dT) beads. For prokaryotic samples, rRNA is removed using a specialized kit that leaves the mRNA. The mRNA from either eukaryotic or prokaryotic sources was then fragmented randomly in fragmentation buffer, followed by cDNA synthesis using random hexamers and reverse transcriptase. After first-strand synthesis, a custom second-strand synthesis buffer (Illumina) is added, with dNTPs, RNase H, and *Escherichia coli* polymerase I to generate the second strand by nick-translation, and AMPure XP beads are used to purify the cDNA. The final cDNA library is ready after a round of purification, terminal repair, A-tailing, ligation of sequencing adapters, size selection, and PCR enrichment. Library concentration was first quantified using a Qubit 2.0 fluorometer (Life Technologies) and then diluted to I ng/gl before checking insert size on an Agilent 2100 and quantifying to greater accuracy by quantitative PCR (Q-PCR) (library activity >2 nM). Libraries are fed into Novaseq6000 machines according to activity and expected data volume.

### RNA-seq analysis

Differential expression of genes between AdipoRon-treated group and AdipoRonPEG5-treated group was analyzed by use of genes having an fpkm of ≥0 in all samples. Genes whose expression was “between 1.1 and 0.9 fold change” or “more than 1.1 fold up- “or “less than 0.9 fold down-“ regulated in liver RNA from control (*n* = 3) versus AdipoRon (*n* = 3) versus AdipoRonPEG5 group constituted “nc”, “up,” and “down” signatures. Gene expression was analyzed using Gene Ontology analysis (GO Consortium) and GenePattern software (Broad Institute).

### Sphingolipid isolation from tissues

Flash-frozen tissue samples (~40 mg) in a borosilicate glass tube were quenched with 2.0 ml of organic extraction solvent (isopropanol: ethyl acetate, 15:85; v:v) and homogenized with a mechanical tissue disruptor. Immediately afterward, 20 μl of organic internal standard solution was added (Ceramide/Sphingoid Internal Standard Mixture II diluted 1:10 in ethanol). The mixture was vortexed for 20 s and sonicated in ultrasonic bath for 40 min at 40°C. The organic extract was allowed to reach room temperature, and 1.5 ml of HPLC water was added and a two-phase liquid-liquid extraction was performed. The upper phase was transferred to a new clean tube, and the lower aqueous phase containing the protein pellet was re-extracted with additional 2.0 ml of organic extraction solvent. The organic phases were combined and dried under nitrogen stream at 40°C. Dried extracts were stored at –80°C until analysis. For analysis of ceramides and sphingoid base species, the dried residues were reconstituted in 200 μl of methanol. Sphingomyelins required a sample dilution of 1:50 in methanol prior to analysis. Serum sphingolipids were extracted using a similar methodology requiring 25 μl of serum.

Liver, subcutaneous adipose tissue, pancreas, pancreatic islets were initially homogenized in 500 μl of cold PBS using an ultrasonic probe disruptor and keeping the sample on ice during sonication. In total, 50 μl of aqueous homogenate was aliquoted for protein determination. In total, 400 μl of the lysate was immediately quenched in 2.0 ml of organic extraction solvent.

### Sphingolipid measurement and quantitation

In total, 5 μl of reconstituted samples was injected into an LC/MS/MS system for the analysis of ceramides and sphingoid bases, and 1 μl injection was required for the analysis of sphingomyelins. The system consisted of a Shimadzu LCMS-8050 triple-quadrupole mass spectrometer with the dual ion source operating in electrospray positive ionization mode. The mass spectrometer is coupled to a Shimadzu Nexera X2 UHPLC system equipped with three solvent delivery modules LC-30AD, three degassing units DGU-20A5R, an autosampler SIL-30ACMP, and a column oven CTO-20AC operating at 40°C (Shimadzu Scientific Instruments, Columbia, MD). Six sphingoid bases (d18:1 sphingosine, d18:1 sphingosine-1-phosphate, d18:1 deoxysphingosine, d18:0 sphinganine, d18:0 sphinganine-1-phosphate, d18:0 deoxysphinganine) and 17 ceramide species and their metabolites (C16:0, C16:0 dihydroceramide, C16:0 glucosylceramide, C16:0 lactosylceramide, C16:0 sphingomyelin, C18:0, C18:0 dihydroceramide, C18:0 glucosylceramide, C18:0 sphingomyelin, C18:1, C20:0, C22:0, C22:0 glucosylceramide, C24:0, C24:0 dihydroceramide, C24:0 lactosylceramide, C24:0 sphingomyelin, C24:1, C24:1 dihydroceramide, C24:1 glucosylceramide, C24:1 sphingomyelin). Quantitative analysis of sphingolipids was achieved using selective reaction monitoring scan mode. Lipid separation was achieved by reverse phase LC on a 2.1 (i.d.) × 150 mm Ascentis Express C8, 2.7 micron (Supelco, Bellefonte, PA) column under gradient elution, using three different mobile phases: eluent A consisting of methanol/water/formic acid, 600/400/0.8, v/v/v with 5 mM ammonium formate, eluent B consisting of methanol/formic acid, 1,000/0.8, v/v with 5 mM ammonium formate, and eluent C consisting of CH_3_OH/CH_2_Cl_2_ 350/650. The concentration of each metabolite was determined according to calibration curves using the peak-area ratio of analyte versus corresponding internal standard. Calibration curves were generated using serial dilutions of each target analyte.

### Ceramidase activity assay

INS1 cells were cultured in 10 cm dish and treated with AdipoRon or AdipoRonPEG5 (20 μM) for 24 h. Remove the media and collect cells using a cell scraper and transfer cells to a 1.5 ml tube. Centrifuge to spin down the cells before freezing in liquid nitrogen. Cell pellets were stored at –80°C till analysis. During analysis, INS1 cell pellets on dry ice were mixed with 500 μl of Dulbecco PBS with Ca^2+^ and Mg^2+^. The sample was then immediately placed on ice and lysed with a tissue disruptor probe (Fisher Scientific, Model FB50, Waltham MA) by performing five short disruption cycles at a power of 30% (3 s each cycle). Briefly perform a gentle vortexing of the vial. Take an aliquot of 50 μl of sample for protein determination (Pierce™ BCA Protein Assay Kit, Thermo Fisher Scientific). In total, 400 μl of lysate was transferred to a culture borosilicate glass tube containing 1.6 ml of Dulbecco PBS with Ca^2+^ and Mg^2+^ on ice. In total, 20 μl of labeled ceramide cocktail is added to the glass tube containing the lysate (10 μM concentration of each component of the mixture in ethanol: Ceramide-d7(d18:1/16:0), Ceramide-d7(d18:1/18:0), Ceramide-d7(d18:1/24:0), Ceramide-d7(d18:1/24:1), Avanti Polar Lipids, Alabaster, AL). Lysates were vortexed briefly and incubated at 37°C in a shaker incubator during 2 h. After incubation samples were quenched by adding an organic extraction solvent (2.0 ml, AcOEt/IPA; 85:15, v:v) and vortexed. In total, 20 μl of internal standard mixture is added (Sphingolipids Mixture I without ceramide (d18:1/25:0), diluted 1:10 in ethanol, Avanti Polar Lipids). Two-phase liquid-liquid extraction cycles were performed, organic phases are combined and dried down under a stream of nitrogen with heating at 40°C. Dry extracts were reconstituted in 200 μl, and serial dilutions were prepared at 1:5 and 1:50 dilution of the first reconstituted extract. All the samples and serial dilutions were analyzed by injecting 1 μl in the LC-MS/MS system: Shimadzu LCMS-8060 triple-quadrupole mass spectrometer coupled to a Nexera X2 UHPLC chromatograph (Shimadzu Scientific Instruments, Columbia, MD) operating the dual ion source (DUIS) in electrospray positive ionization mode. Compounds were separated on a Ascentis® Express C18 UHPLC column (150 × 2.1 mm, 2.7 μm HPLC Column, Supelco, Bellefonte, PA) by using a gradient of 5 mM ammonium formate 0.8% formic acid (v:v) in water (solvent A) and 5 mM ammonium formate 0.8% formic acid (v:v) in methanol (solvent B) at a flow rate of 0.4 ml/min. LabSolutions V 5.93 and LabSolutions Insight V 3.1 SP1 program packages were used for data processing (Shimadzu Scientific Instruments)

### Statistics

Statistical analysis was carried out following Student's *t* test or analysis of variance test (ANOVA) with Tukey's test using Prism software (Graphpad, San Diego, CA). A *P* value of less than 0.05 was statistically significant. Corresponding significance levels are indicated in the figures. All data are presented as standard error of the mean ± SEM.

## Results

### Enhancing AdipoRon solubility in aqueous environments results in higher bioavailability

**Fig. 1:**
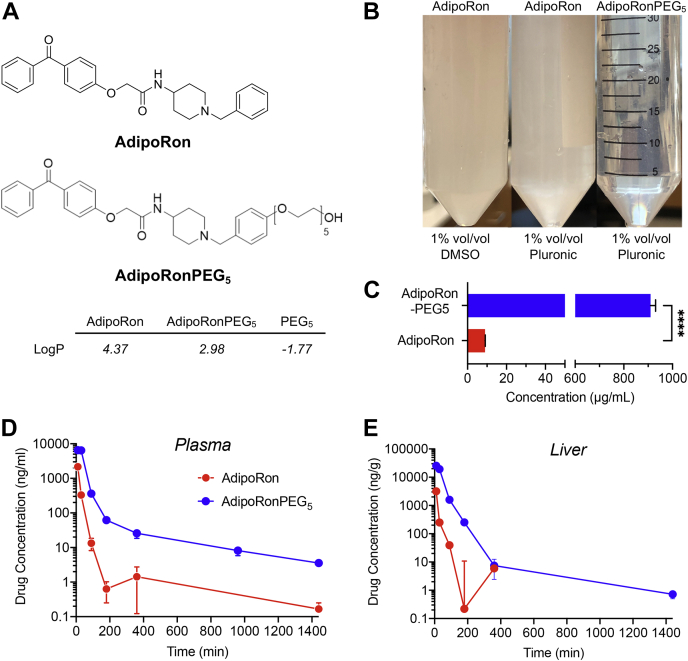
Enhancing AdipoRon water solubility improves pharmacokinetics and bioavailability. A: Chemical structures of AdipoRon and AdipoRonPEG_5_ and their predicted LogP values. B: Images of AdipoRon and AdipoRonPEG5 aqueous solutions (both 1.2 mM) with additives. C: Quantification of water solubilities by LC-MS/MS. D and E: Pharmacokinetic profiles of AdipoRon and AdipoRonPEG_5_ in the plasma (D) or liver (E) upon IP dosing at 20 mg/kg in 8-week-old C57BL/6J mice (N = 3, Mean ± SEM).

AdipoRon is poorly soluble in aqueous media even in the presence of DMSO or a nonionic solubilizer, such as pluronic or Cremophor (Fig. 1A, B). We, therefore, explored the effects of modifying AdipoRon with ethylene glycol units of varying but discrete lengths on its bioavailability in mice and cytoprotective efficacy in INS-1 cells. Polyethyleneglycol (PEG) is a sterically unhindered linker that associates in solution with approximately two water molecules per ethylene glycol unit. The addition of a pentaethylene glycol moiety (PEG_5_) to AdipoRon yielded AdipoRonPEG_5_, which was predicted to be less lipophilic with an octanol-water partition coefficient (logP value) that was 1.4 unit less than that of AdipoRon. We synthesized AdipoRonPEG5 (supplemental data) and confirmed its improved aqueous solubility (Fig. 1B). Quantification by LC-MS/MS showed that AdipoRonPEG_5_ was about 100 times more soluble in water than the parent molecule with a measured solubility of 910 ± 36 μg/ml (mean ± SD) compared with 8.99 ± 0.15 36 μg/ml for AdipoRon (Fig. 1C). This improved hydrophilicity and solubility of AdipoRonPEG_5_ were reflected in an enhanced bioavailability in the circulation after intraperitoneal injection (IP), with threefold increase in the maximal plasma concentration of AdipoRonPEG_5_ compared with AdipoRon (Fig. 1D, plasma C_max_ of 6510.00 ± 684.62 ng/ml for AdipoRon-PEG_5_ vs. 2166.67 ± 195.53 ng/ml for AdipoRon). The difference in the bioavailability of these drugs in the mouse liver was even more dramatic, with AdipoRonPEG_5_ showing eightfold higher bioavailability in the liver (C_max_ 25509.73 ± 1436.44 ng/ml) than AdipoRon (C_max_ 3169.30 ± 1437.09 ng/ml, Fig. 1E). We attribute these improvements to the PEG_5_ moiety, which endows a combination of benefits to the molecule, including enhanced solubility, prolonged circulation, and more efficient cellular uptake.

### AdipoRon treatment mitigates palmitate-induced lipotoxicity in INS-1 β-cells

**Fig. 2:**
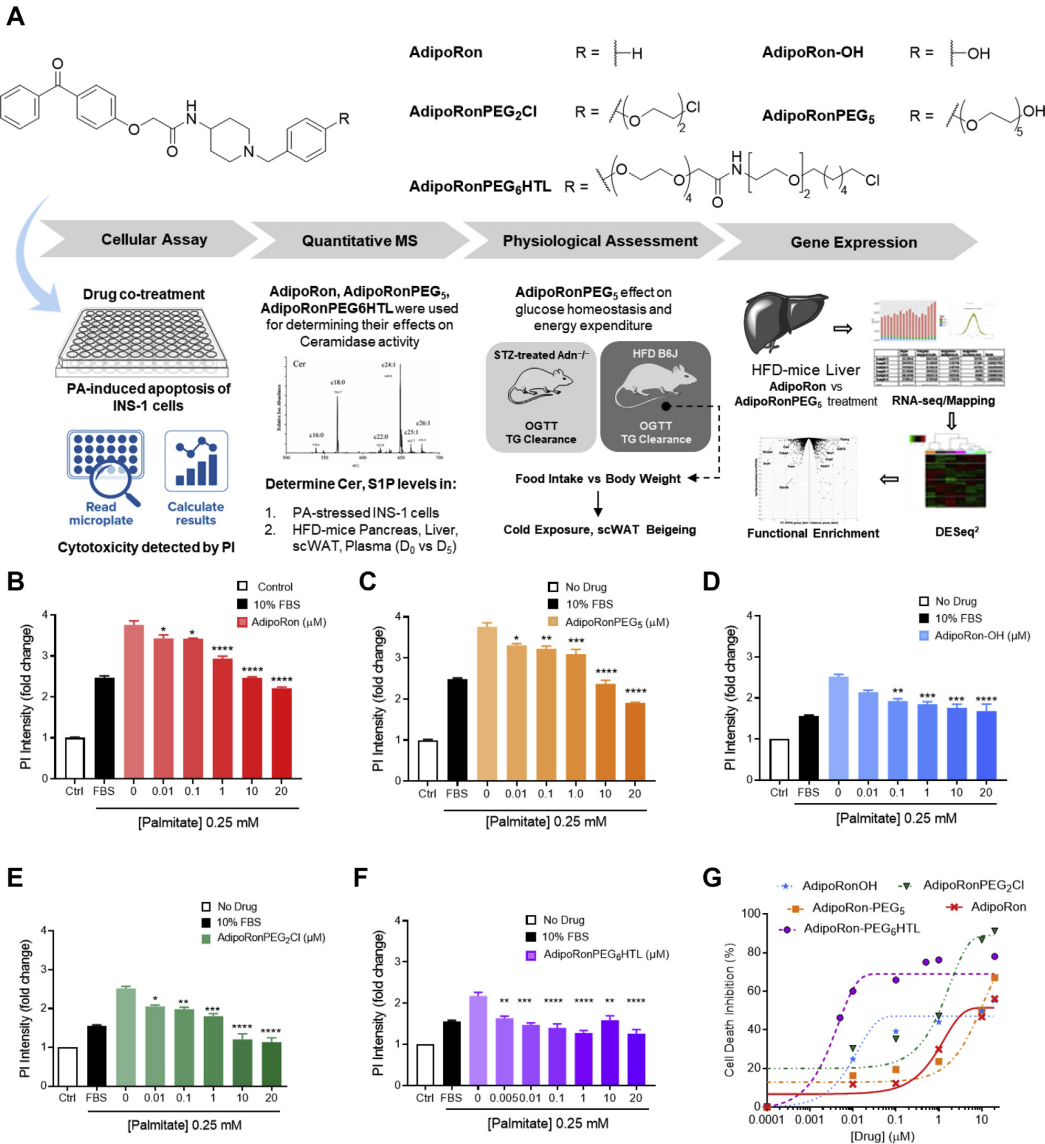
Schematic representation of this work from compound screening to the mechanistic study. A: Experimental scheme of the present study including the structures of AdipoRon Analogs screened using PA-stressed INS-1 cells and quantified after treatment with PI using a platereader. AdipoRon analogs, namely AdipoRon for reference, AdipoRonPEG_5_ and AdipoRonPEG_6_HTL were selected to assess their effect on ceramide levels (Cer) in INS-1 cells and in mouse under HFD conditions (pancreas, liver, scWAT; plasma). This is followed by systematic testing of glucose, lipid and energy expenditure in HFD mouse treated with AdipoRonPEG_5_. A similar treatment on T1D mouse model (STZ-stressed Adn^−^/^−^) assessed the drug effect on insulin sensitivity and lipolysis. Finally, RNA-seq analysis of HFD-mouse liver was implemented to unravel the mechanisms by which AdipoRonPEG_5_ exerts its metabolic effects in the liver. B–F: AdipoRon analogs protect β-cells from palmitate-induced cell death. INS-1 β-cells were treated with different dosages of AdipoRon (B), AdipoRonPEG_5_ (C), AdipoRonOH (D), AdipoRonPEG_2_Cl (E), or AdipoRonPEG_6_HTL (F) for 24 h in the presence of palmitate (0.25 mM). The cells were then labeled with PI (1 mg/ml) for 5 min and measured on a fluorescence plate reader. PI fluorescence intensity was normalized against control cells that were not stressed with palmitate. Results are expressed as mean ± SEM (n = 4). ∗*P* < 0.05; ∗∗*P* < 0.01; ∗∗∗*P* < 0.001, ∗∗∗∗*P* < 0.0001 versus 0 mM of compound. G: Dose-response curves of AdipoRon and derivatives in protecting INS1 cells from palmitate induced cell death.

Palmitic acid (PA), among other nonesterified fatty acids, triggers the activation of proapoptotic signals as well as induces lipotoxicity through the formation of ceramides (38, 39). As a model of β-cell dysfunction, we treated INS-1 β-cells with PA (0.25 mM) for 24 h, then stained the treated cells with Hoechst 33342 and propidium iodide (PI) to distinguish apoptotic from necrotic cells. As a result, a marked increase in the number of apoptotic cells was observed by confocal microscopy (supplemental Fig. S1). These images showed that PA induced β-cell death mainly via apoptosis with a negligible necrotic component under the experimental condition. This assay was used to assess the antiapopotic potential of AdipoRon and its analogs. We synthesized several AdipoRon analogs via the AdipoRon-OH intermediate as described in SI (supplemental Scheme S1 and S2). AdipoRon-OH was thus employed for the synthesis of AdipoRonPEG_2_Cl, AdipoRonPEG_5_, and AdipoRonPEG_6_HTL, where HTL is known as a hexylchloride HaloTag ligand (Fig. 2A).

To quantitatively determine the antiapoptotic potential of AdipoRons in β-cells, we evaluated AdipoRon in INS-1 β-cells using a cell death protection assay (Fig. 2A) (12), where the rescue of PA-induced cell death was assessed after treating cells with AdipoRon analogs at different concentrations. Cell death was quantified from the PI fluorescence intensity using a fluorescence plate reader. When INS-1 β-cells were cultured with PA at 0.25 mM for 24 h in the absence of serum, an approximately fourfold increase in PI fluorescence signal was observed compared with the untreated cells. FBS (10% vol./vol.), used as a positive control, resulted in approximately ~34% reduction in apoptosis compared with cells treated with PA alone. AdipoRon exhibited a similar protection as FBS when used at 10 μM, with a further modest increase in protection at 20 μM (Fig. 2B).

AdipoRonPEG_5_ also exerted a potent antiapoptotic activity when cells were stressed with PA (Fig. 2C). At 20 μM, AdipoRonPEG_5_ reduced PA-induced apoptosis by nearly 50%, surpassing the protective effect from FBS, demonstrating that pegylation of AdipoRon provided the benefit of enhanced solubility without compromising its biological activity. With this encouraging result in hands, we opted to investigate the effect of PEG linkers on the activity of AdipoRon derivatives systematically. We first compared AdipoRon-OH with AdipoRonPEG_2_Cl. While both analogs showed similar protective activity up to 1 μM, cells treated with AdipoPEG_2_Cl at 10 μM showed close to 90% viability protection from PA-induced apoptosis (Fig. 2D, E). We hypothesize that having an amphiphilic PEG linker enhances AdipoRon solubility in aqueous environments without compromising its cell permeability. To test our hypothesis further, AdipoRonPEG_12_, AdipoRonPEG_6_HTL, and AdipoRonPEG_13_HTL activities were assessed in a similar way. We envision using HTL-modified AdipoRon for the future targeted delivery and enrichment of AdipoRon to β-cells that are transgenically modified to express a HaloTag (40, 41, 42) with the goal of bypassing systemic activation of AdipoR, which could pose certain risks to subjects and patients (43, 44). With that in mind, the dose-dependent antiapoptotic activity of AdipoRonPEG_6_HTL was determined. Notably, a significant inhibition of PA-induced cell death was observed at all concentrations of AdipoRonPEG_6_HTL, reaching almost a complete inhibition even at 0.01 μM (Fig. 2F, G). In contrast, when AdipoRon was modified with a relatively long polydispersed ethylene glycol chain as in the case of AdipoRonPEG_12_ (supplemental Fig. S2B), the protective effect of AdipoRon was substantially diminished. This may be caused by the increased hydration effect of the longer ethylene glycol chain, which could lead to an effective change in the surrounding environment, hence altering AdipoRon affinity to AdipoR1/2. In contrast, AdipoRonPEG_13_HTL, having a hydrophobic hexylchloride chain in addition to the ethylene glycol linker, retains its cytoprotective effect in INS-1 cells stressed with PA (supplemental Fig. S2C). This suggests that it is important to balance the amphiphilicity of these pegylated AdipoRon analogues to maintain the cytoprotective activity. Furthermore, these results suggest that AdipoRon analogs may be used in vivo at lower concentrations than the parent molecule, possibly mitigating any potential side effects.

### AdipoRon analogs lower ceramide levels in PA-stressed INS-1 β-cells

**Fig. 3:**
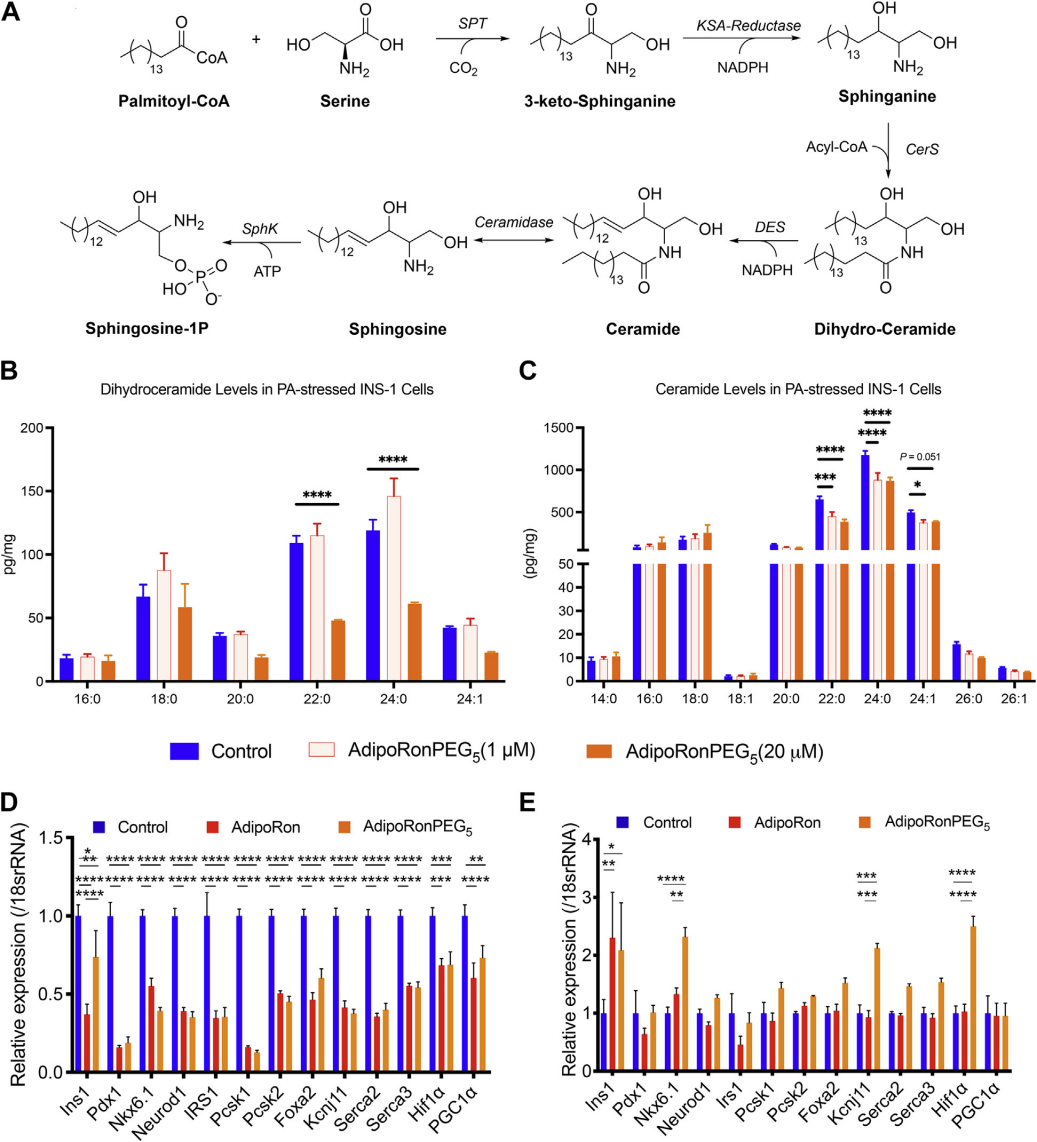
AdiporonPEG_5_ reduces β-cell ceramide species and promotes expression of β-cell identity genes in the presence of palmitate. A: De novo synthesis of sphingolipids begins with the condensation of serine and palmitate to 3-ketosphinganine, which is reduced to sphinganine. Ceramide synthase catalyzes the N-acylation of sphinganine to dihydro-ceramide, which forms ceramide after desaturation. B and C: Changes in the cellular levels of dihydro-ceramide (B) and ceramides (C) in response to drug treatment. INS1 cells treated with palmitate (0.25 mM) and drugs for 24 h were assayed for their ceramide levels by mass spectrometry. Changes in the lipid level were normalized against control cells treated with palmitate. D and E: Relative gene expressions of insulin secretion related genes in INS-1 cells treated with AdipoRon or AdipoRonPEG_5_ (both 20 μM) in the absence of palmitate (D) or in the presence of palmitate (E) (n = 6). Data are mean ± SEM. ∗*P* < 0.05, ∗∗*P* < 0.01 and ∗∗∗*P* < 0.001.

Ceramides are bioactive lipids whose accumulation has been shown to lead to β-cell cytotoxicity through contributing to death receptor clustering, apoptosome formation, and Bcl-2-associated X protein (Bax) translocation (45). The biosynthesis of ceramides, illustrated in Fig. 3A, shows that ceramides can be interconverted into sphinogosine-1 phosphate (S1P) via the intermediate product sphingosine (Sph). In contrast to ceramides, both S1P and Sph promote survival, nutrient uptake, nutrient utilization, and mitochondrial proliferation by increasing intracellular calcium and activate AMPK through the stimulation of calcium/calmodulin-dependent protein kinase kinase, CAMKK (46, 47). In leptin-deficient (*ob/ob*) mice, adiponectin stimulates ceramidase activity via its receptors, thus lowering hepatic ceramide levels (12). To determine whether AdipoRon analogs exert the same ceramidase effect on INS-1 cells, we employed mass spectrometry to determine the levels of ceramides levels after 24 h incubation with AdipoRonPEG_5_ in the presence of 0.25 mM PA. AdipoRonPEG_5_ markedly lowered overall dihydroceramide and ceramides levels (Fig. 3B, C and supplemental Table S4). Particularly, C22:0 and C24:0 ceramides were significantly reduced using AdipoRonPEG_5_ even at 1 μM, demonstrating its potent effect on ceramide reduction in INS1 cells.

Gene expression analysis of INS-1 cells in the absence of palmitate by qPCR revealed that both AdipoRon and AdipoRonPEG_5_ suppress genes related to insulin secretion and transcription factors that are involved in pancreatic β-cell differentiation (Fig. 3D). This is consistent with reports describing the downregulation of insulin secretion genes upon phosphorylation of AMPK, a direct effect of AdipoR activation (48, 49, 50). Additionally, qPCR data shows the downregulation of pancreatic and duodenal homeobox gene 1 (Pdx-1) and other genes involved in β-cell maintenance. In sharp contrast, in the presence of palmitate, AdipoRonPEG_5_ significantly increased the expression of genes that are involved in maintaining the functional state of pancreatic β-cell, notably *Ins1*, *Nkx6-1*, and *Kcnj11* (Fig. 3E) (51). Furthermore, AdipoRonPEG_5_ treatment of PA-stressed INS-1 cells elevates the expression of *hypoxia-inducible factor-1α* (*Hif-1α*), a protective gene that can improve β-cell function and prevent environmentally triggered T1D (52). These observations suggest that AdipoRonPEG_5_, compared with AdipoRon, exerts enhanced protective actions against PA-mediated β-cell cytotoxicity and seems to add further improvements toward maintaining the functional integrity of β-cells under these conditions.

### AdipoRonPEG_5_ exerts pleiotropic actions in mice under insulinopenic and other metabolic challenging conditions

**Fig. 4:**
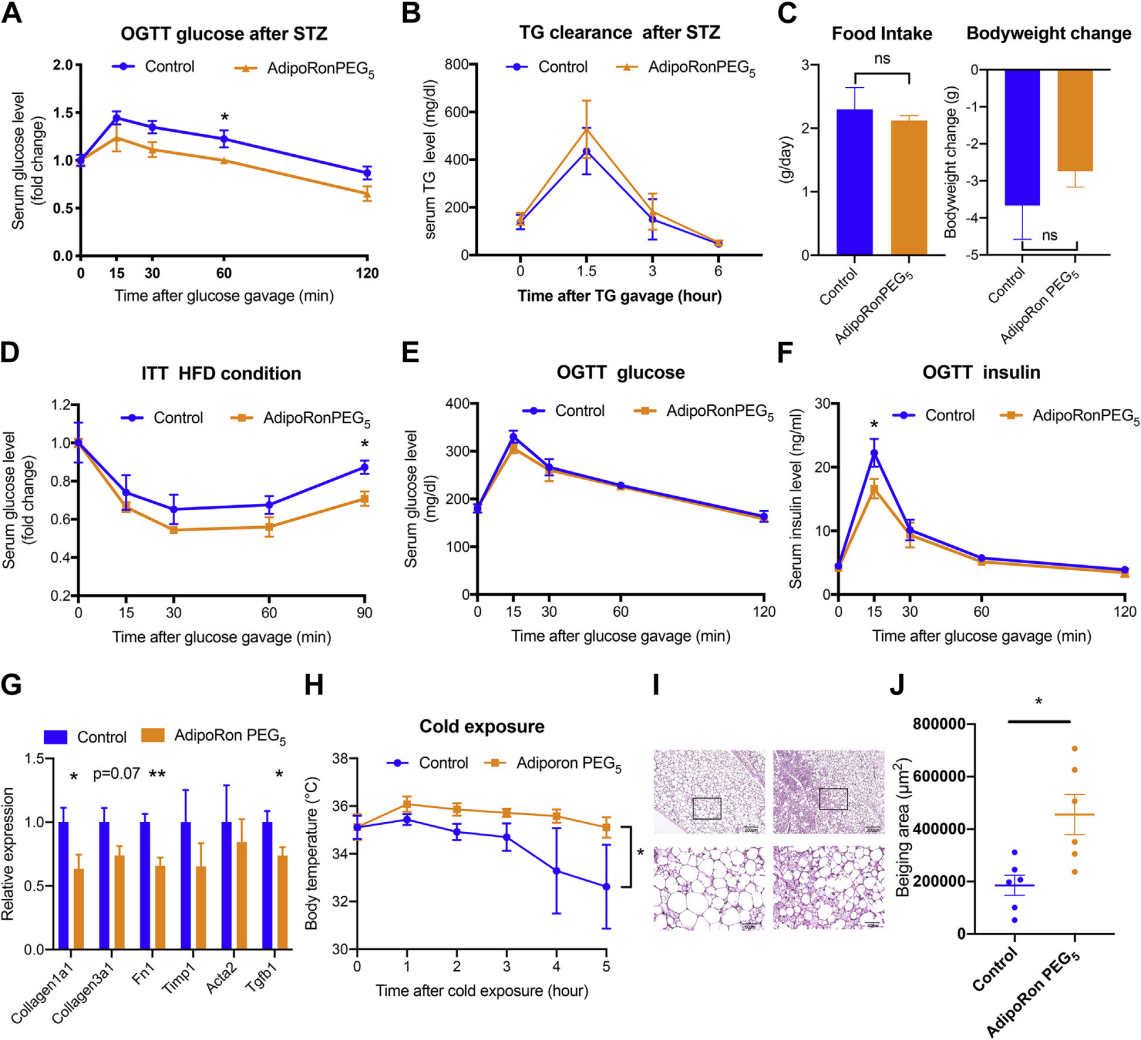
AdipoRonPEG_5_ improved glucose metabolism under insulinopenic and high-fat diet conditions and promotes adipocyte beiging after cold exposure. A and B: Adiponectin-deficient mice were treated with streptozotocin to deplete insulin-producing β-cells followed by oral glucose tolerance test (A) and triglyceride tolerance test (B) (n = 8). C: Wild-type mice were fed high-fat diet for 7 months and treated with AdipoRonPEG_5_ (5 mg/kg) twice a day for 5 days. Food intake and body weight were measured before and after the treatment (n = 4 control and n = 5 AdipoRonPEG_5_). D: Blood glucose levels of mice under HFD were measured after intraperitoneal insulin insulin injection (1.25 mU/g body weight) (n = 4 control and n = 5 AdipoRonPEG_5_). E and F: Blood glucose and insulin level were determined after oral glucose gavage (2.5 g/kg body weight) (n = 4 control and n = 5 AdipoRonPEG_5_). G: Fibrosis-related genes were measured by qPCR. (n = 9 control and n=10 AdipoRonPEG_5_) (H) Body temperature of the mice treated with AdipoRonPEG_5_ (5 mg/kg) was determined after cold exposure (4°C) (n = 6). I: Representative HE stain images of control (left panel) and AdipoRonPEG_5_ (right panel) treated subcutaneous adipose tissue after cold exposure. J: Quantification of multilocular area of subcutaneous adipose tissue (n = 6). Data are mean ± SEM. ∗*P* < 0.05, ∗∗*P* < 0.01.

Although AdipoRonPEG_6_HTL showed good potential for lowering ceramide levels in INS-1 cells (Fig. 2F, G), a dose of 5 mg/kg injected twice daily in mice on HFD over 5 days resulted in rapid weight loss and necessitated early termination of mice (supplemental Fig. S3A, B). Consequently, we were unable to compare the treatments of AdipoRon and AdipoRonPEG_6_HTL using the same dose. Instead, given its cytoprotective effect and enhanced solubility, we shifted our focus to AdipoRonPEG_5_ to assess its effect in vivo using the insulinopenic adiponectin knockout mouse (Adn^−/−^) or HFD-induced obese mice. In STZ-treated Adn^−/−^ mice, exogenous adiponectin suppresses lipolysis in adipose tissue under insulinopenic conditions (14). By comparison, while AdipoRonPEG_5_ treatment improved the glucose levels as determined by an OGTT (Fig. 4A), blood TG levels were not altered (Fig. 4B). It is yet to be investigated whether the glucose homeostasis observed upon AdipoRonPEG_5_ treatment is the result of improved insulin sensitivity and/or AdipoRonPEG_5_'s protective role against STZ-induced β-cell apoptosis.

To evaluate the effect of AdipoRonPEG_5_ on insulin sensitivity of a T2D mouse model, we fed mice with HFD for 7 months followed by AdipoRonPEG_5_ treatment (5 mg/kg) twice a day for 5 days. AdipoRonPEG_5_ treatment at this dosage had no significant impact on food intake or body weight (Fig. 4C), confirming no overt toxicity toward overall physiology, hence allowing us to assess its beneficial effect on lipid and glucose metabolism. ITTs showed a significant reduction of blood glucose levels (Fig. 4D). On the other hand, OGTTs showed that blood glucose levels were not altered, yet the OGTT was accompanied by a reduction of blood insulin levels, suggesting that the insulin sensitivity was improved upon AdipoRonPEG_5_ treatment (Fig. 4E, F).

To assess the mechanism by which AdipoRonPEG_5_ improved insulin sensitivity or exerted adiponectin-like effects in vivo, we evaluated the expression of adiponectin-regulated genes in the pancreas, brown adipose tissue (BAT) and scWAT of mice on HFD. Although there is a possibility that AdipoRonPEG_5_ is directly acting on β-cells and conveys beneficial effects for survival, at least under HFD conditions, we did not detect a significant upregulation of genes that are involved in maintaining the functional state of pancreatic β-cells when assaying the whole pancreas lysate (supplemental Fig. S4A). On the other hand, there was a significant upregulation of *Prdm16* (a transcription factor that regulates the thermogenic gene program in BAT) and to lesser extent of BAT-specific thermogenesis genes, such as *cell death inducing DFFA like effector* a (*Cidea*) and *Uncoupling protein1* (*Ucp1*) (*P* = 0.06, 0.08, respectively) (supplemental Fig. S4B). AdipoRonPEG_5_ treatment also resulted in increased expression of Cpt-1a, which encodes the mitochondrial outer-membrane-bound enzyme *carnitine palmitoyltransferase* (*Cpt-1a*). Cpt-1a contributes to mitochondrial fatty acid β-oxidation (FAO) via esterification of free fatty acids with CoA, which are then transferred to the mitochondrial matrix to generate acetyl-CoA (53). Interestingly, overexpression of an active Cpt-1a was able to ameliorate insulin resistance in mice (53) which could also be associated with the phenotype that was observed in AdipoRonPEG_5_-treated mice (supplemental Fig. S4B).

Gene expression analysis of scWAT was performed by utilizing scWAT derived from two groups: short-term HFD (4 weeks) and long-term HFD (7 months). Gene expression of scWAT indicated a possible antifibrotic effect of AdipoRonPEG_5_ that mirrors the actions of adiponectin on scWAT. In particular, adiponectin exerts potent regulatory effects on fibroblasts by inhibiting profibrotic responses induced by *transforming growth factor beta1* (*Tgfb1*) (54). Adiponectin's antifibrotic effects are mediated by the activation of AMPK that results in the downregulation of fibrotic genes. Consistent with adiponectin's impact on the fibrosis program, AdipoRonPEG_5_ treatment lowered fibrosis-related genes (*Col1a1*, *Col3a1*, and *Fn1*) accompanied by lower *Tgfb1* expression in subcutaneous adipose tissue of HFD mice (Fig. 4G). Through evaluating the impact of AdipoRonPEG_5_ under short-term and long-term HFD separately, consistent reduction of fibrosis-related genes was observed in both groups (supplemental Fig. S5A, B). These observations are consistent with results from other groups (55) reporting that AdipoRon mitigates HFD activation of subcutaneous fibrosis, further highlighting the wide spectrum of beneficial actions of AdipoRon and its analogs.

As adiponectin is an important regulator of energy homeostasis (18, 55) and its major downstream target AMPK improves glucose metabolism through the activation of brown and beige adipocytes (56), we also assessed the adaptive cold-induced thermogenesis in wild-type mice upon treatment with AdipoRonPEG_5_. After 5 days of AdipoRonPEG_5_ treatment, we performed acute cold exposure experiments and assessed the body temperature. In accordance with established adiponectin readouts, the AdipoRonPEG_5_-treated group maintains significantly higher body temperature (Fig. 4H). Histological analysis reveals that AdipoRonPEG_5_ subcutaneous adipose tissue shows significant structural changes, including the formation of polygonal cells containing several small lipid droplets, resembling beige adipocytes (Fig. 4I, J). The phenotype of brown-in-white or “brite” adipocytes was also significantly evident based on the qPCR results of T-cadherin (CDH13) (supplemental Fig. S6A), while there was no significant overexpression of browning-related transcription factors, such as PPAR-γ, nor of UCP-1, the thermogenesis effector. Similarly, aside from the upregulated expression of CDH13, qPCR analysis of thermogenesis-related genes in BAT did not show any significant increases that reflect the improved adaptive cold-induced thermogenesis (supplemental Fig. S6B). The expression of surface protein T-cadherin, which has adiponectin binding properties, has been observed in M2 macrophages that accumulate via self-renewal in the stromal vascular fraction (SVF) of scWAT in response to chronic cold exposure, a process that has been proposed to result from the T-cadherin-dependent sequestration of adiponectin in the SVF (18, 57). Our qPCR data suggest that AdipoRonPEG_5_ treatment contributed to the upregulated expression of T-cadherin; and the observed functional improvements in beige and brown adipose tissue may be explained via posttranscriptional activation of the classical thermogenic pathway or through the activation of one of the more recently described alternative pathways, such as the creatine-driven substrate cycle (58).

### AdipoRonPEG_5_ activate adiponectin signaling pathways including ceramidase and AMPK

To investigate the mechanisms that improve insulin sensitivity in response to AdipoRonPEG_5_-treatment of HFD mice, we assessed the activation of the AMPK and ceramidase pathways and the levels of long-chain ceramides known to have cytotoxic effects that adversely affect lipid metabolism in the liver, pancreas, and subcutaneous adipose tissue (59).

**Fig. 5:**
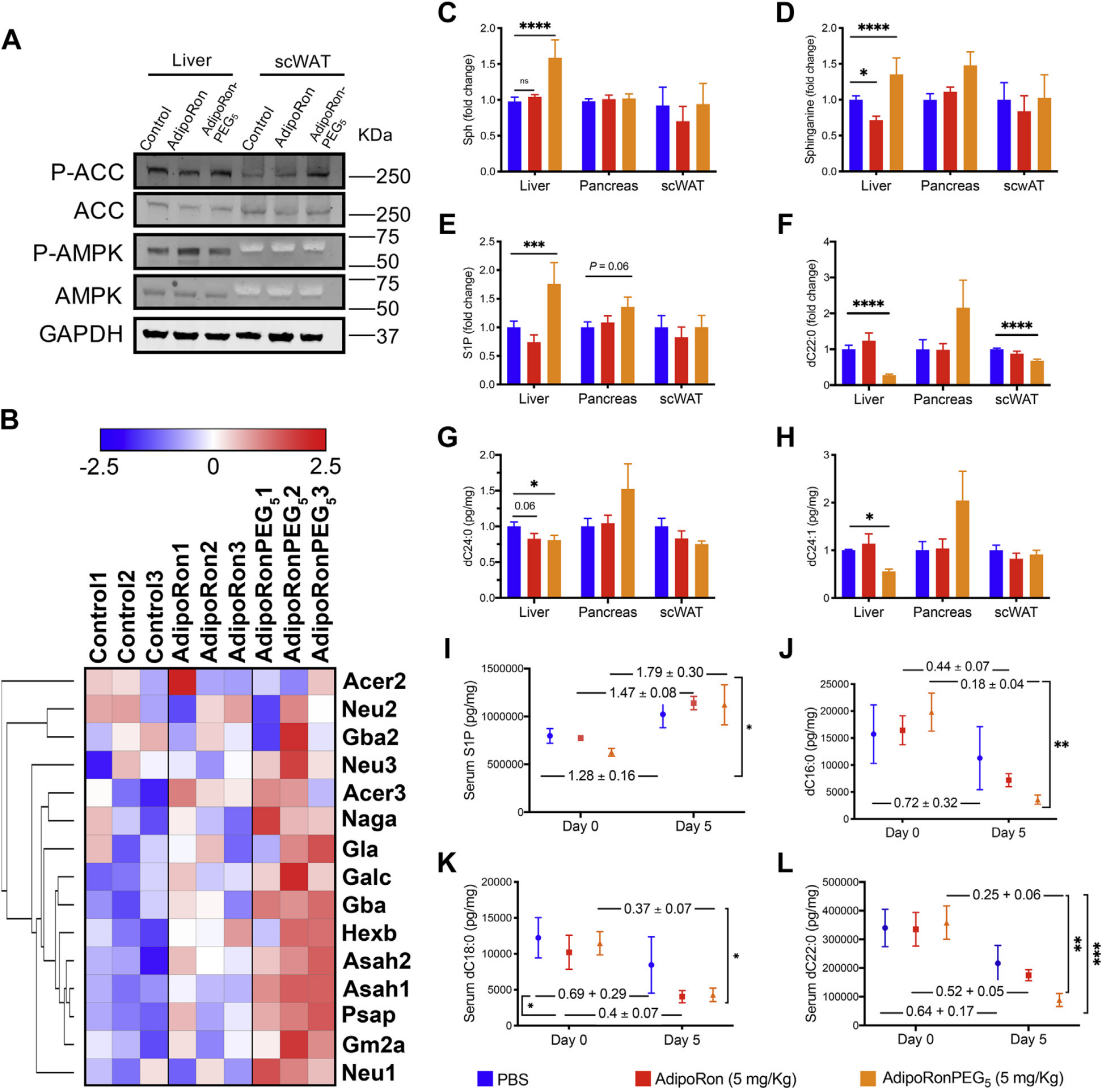
AdipoRonPEG_5_ activates AMPK signaling and reduces ceramide species in vivo. A: Liver and subcutaneous adipose tissue were harvested after single treatment of AdipoRon or AdipoRonPEG_5_. Representative images of the western blot for phosphorylation of AMPK and ACC (n = 4). B–L: Wild-type mice were fed high-fat diet for 4 weeks and treated with AdipoRon or AdipoRonPEG5 (5 mg/kg) twice a day for 5 days before harvesting tissues. RNAseq of liver derived RNA and mass spectrometry analysis of ceramides were performed. B: Heatmap of the genes involved in ceramidase activity. C–L: Sphingoid bases and dihydroceramide species fold-change levels in the liver, pancreas, subcutaneous adipose tissue (C–H), and serum (I–L, fold-change between D0 and D5 were indicated on the graph) were determined by mass spectrometry (n = 5). Data were analyzed by two-way ANOVA with Tukey's test. Data are mean ± SEM. ∗*P* < 0.05, ∗∗*P* < 0.01, ∗∗∗*P* < 0.001 and ∗∗∗∗*P* < 0.0001.

Consistent with the report that AdipoRon enhanced AMPK phosphorylation (23), AdipoRonPEG_5_ also activated the AMPK pathway in the liver and enhanced acetyl CoA carboxylase (ACC) phosphorylation in the subcutaneous adipose tissue (Fig. 5A). Phospho-AMPK was more difficult to visualize, particularly in adipose tissue, while some activation could be observed in the liver. These data support the involvement of canonical adiponectin signaling pathways in mediating the effects of AdipoRonPEG_5_. Moreover, since AdipoRonPEG_5_-promoted phosphorylation of ACC was suppressed in AdipoR2 KO and AdipoR1/AdipoR2 double KO (global AdipoR2 KO/Adiponectin rtTA::TRE-Cre::AdipoR1 flox, unpublished) subcutaneous adipose tissue both in vivo and in vitro (data not shown), we conclude that the signal transduction by AdipoRonPEG_5_ is mediated at least in part through AdipoR1 and AdipoR2.

To gain further insights on the modes of action of AdipoRon and AdipoRonPEG_5_, we proceeded to profile the liver transcriptome by RNA-seq. RNA was extracted from liver tissues of HFD-fed mice for 4 weeks followed by AdipoRon or AdipoRonPEG_5_ treatment (5 mg/kg) for 5 days. From the RNA-seq analysis, we first examined genes involved in ceramide catabolism in the liver of mice under HFD conditions. These include *alkaline ceramidase 2,3* (*Acer2*, *Acer3*), *acid ceramidase* (*N-Acylsphingosine Amidohydrolase 1 Asah1*), among others. A heat map analysis of this subset of genes revealed that the upregulation of genes related to the family of ceramidases was the highest in the AdipoRonPEG_5_ treatment group and to a lesser extent with AdipoRon treatment (Fig. 5B). In order to confirm that the reduction of ceramide is dependent on the increased ceramidase activity, we performed ceramidase activity assay. We incubated AdipoRon or AdipoRonPEG_5_-treated INS1 cell lysate in the presence of labeled ceramides (ceramide16:0-d7, ceramide18:0-d7, ceramide24:0-d7, ceramide24:1-d7) and measured the amount of labeled ceramide levels. In line with the result of RNA-seq analysis, AdipoRonPEG_5_ reduced levels of labeled ceramides, supporting higher level of ceramidase activity upon AdipoRonPEG_5_ treatment (supplemental Fig. S7A–F). In striking contrast, the ceramidase activity in the cell lysate of INS1 cells treated with AdipoRon remained essentially the same as untreated control, demonstrating the superior efficacy of AdipoRonPEG_5_ in promoting ceramidase activity.

MS-based quantification of sphingoid bases (Fig. 5C–E) and ceramides species (Fig. 5F–H) revealed an overall increase of sphingoid bases, including S1P, in the liver of mice under HFD that were treated with AdipoRonPEG_5_. Parallel to this elevation, hepatic dihydroceramides were drastically reduced upon AdipoRonPEG_5_ treatment, especially in the case of dihydroceramide dC22:0, dC24:0, and dC24:1 (Fig. 5F–H, supplemental Tables S5–S8). Such long-chain fatty-acid-containing dihydroceramides have been reported being a good predictor of diabetes development in both human and mouse (60). In contrast to AdipoRonPEG_5_, IP-injected AdipoRon (5 mg/kg) neither increased sphingoid bases nor did it lower dihydroceramide in the liver.

In the pancreata of mice fed with HFD, AdipoRonPEG_5_ had a minimal impact on dihydroceramide levels (Fig. 5F–H), whereas C24:0 and C24:1 ceramides showed a significant reduction upon AdipoRonPEG_5_ treatment compared with the saline controls or to mice treated with AdipoRon (supplemental Fig. S9). In parallel with its effect on ceramide reduction, AdipoRonPEG_5_ treatment also altered S1P level in the mouse pancreas, with a trend of elevating S1P (Fig. 5E). These results highlight the improved efficacy of AdiporonPEG_5_ over AdipoRon to reduce HFD-induced lipotoxicity in the pancreas through an improvement in lipid metabolism, as shown by the ratio of ceramide to sphingosines. It is worth mentioning that another AdipoRon analogue, AdipoRonPEG_6_HTL (Fig. 2A), at 2.5 mg/kg and 0.25 mg/kg induced an overall reduction of pancreatic C14:0, C18:0, C20:0, C24:0, and C24:1 (supplemental Fig. S9A–F). Additionally, pancreatic sphingosine and S1P levels were significantly higher upon treatment with AdipoRonPEG_6_HTL (supplemental Fig. S10A–D). These results are in line with previous reports that adiponectin lowered ceramide levels and elevated S1P concentrations in different tissues, further supporting the role of adiponectin and AdipoR agonists in initiating a receptor-mediated activation of ceramidase activity (12, 26, 61).

In scWAT, neither AdipoRon nor AdipoRonPEG_5_ had a noticeable impact on the ceramide levels (Fig. 5F–H). However, it should be noted that lower quantities of these ceramides were detected in scWAT than in the liver using MS. In comparison, serum dC16:0, dC18:0, dC22:0, and dC24:0 levels were drastically lowered in the AdipoRonPEG_5_ treatment group (Fig. 5I, L). Similarly, IP delivery of AdipoRon also reduced serum dihydroceramides, albeit to a lesser extent than AdipoRonPEG_5_. Together, these data provide a compelling body of evidence to support the notion that AdipoRonPEG_5_ improves lipid and glucose metabolism through the adiponectin signaling pathways that involve both AMPK and ceramide metabolism. The improved bioavailability of AdipoRonPEG_5_ may account for, at least in part, its favorable property in regulating S1P and ceramide levels and its activity in resolving the metabolic syndrome phenotype in HFD mice.

### AdipoRonPEG_5_ contributes to the regulation of glucose and lipid metabolism in the liver

**Fig. 6:**
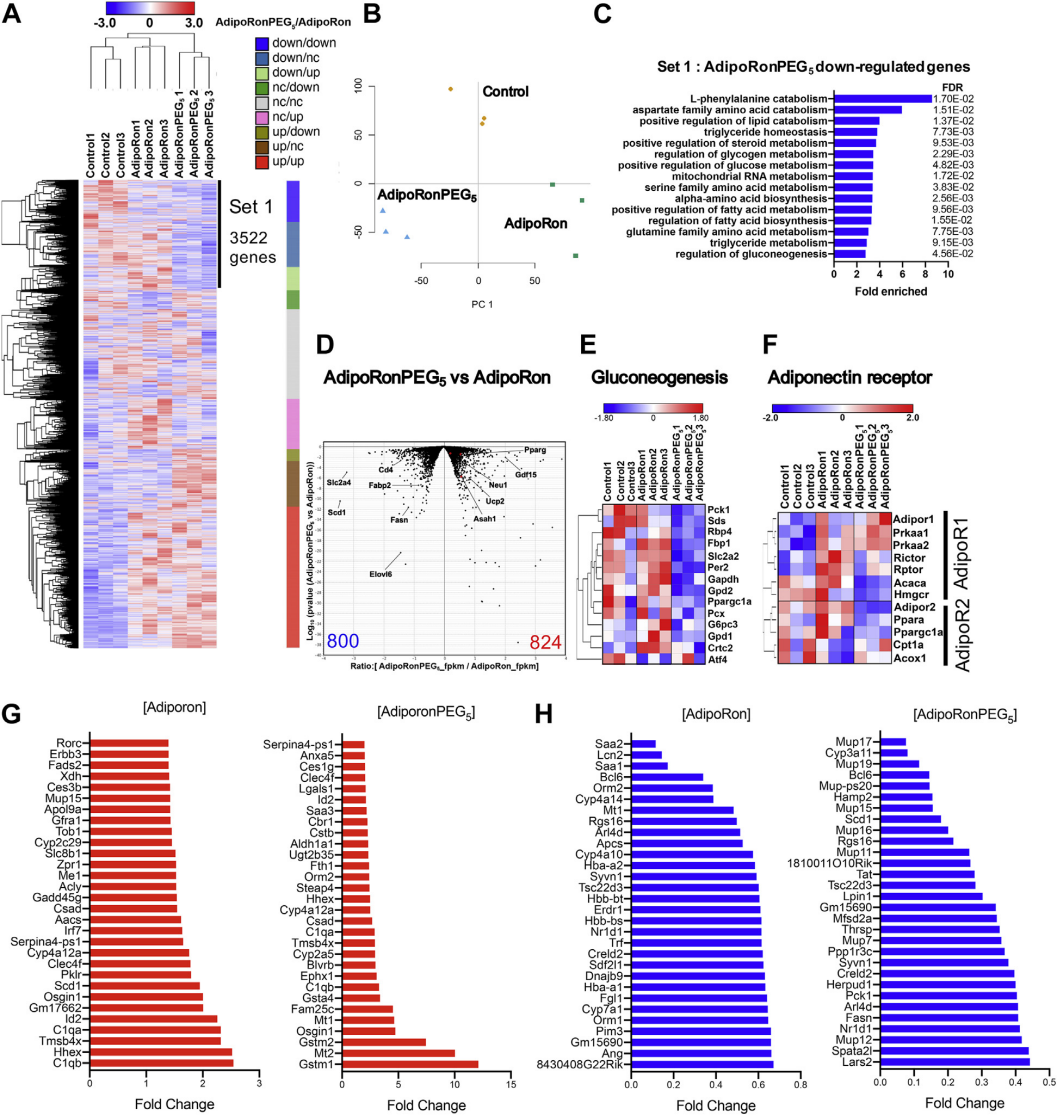
AdipoRonPEG_5_ contributes to the regulation of glucose and lipid metabolism in the liver. A–C: Wild-type mice were fed high-fat diet for 4 weeks and treated with AdipoRonPEG_5_ (5 mg/kg) twice a day for 5 days before the harvest liver. One or two samples were pooled for 1 RNAseq sample in each group. A: Hierarchical clustering of transcriptional profiles in AdipoRon and AdipoRonPEG_5_-treated liver. The genes were divided into nine groups dependent on the response of gene expression to AdipoRonPEG_5_ and AdipoRon treatment. Three kinds of response (up, nc and down) and two kinds of treatment produce nine groups. The criteria of grouping are as follows. Down: fold change < 0.9, nc: 0.9 < fold change < 1.1, up: fold change > 1.1. Right column exhibits the proportion of each group. B: Principal component analysis of whole transcriptomes from all samples. C: 3522 AdipoRonPEG_5_ specifically downregulated genes (Set 1) were identified dependent on hierarchical clustering. Fifteen most enriched pathway by gene ontology (GO) pathway analysis shows the characteristics of genes that were specifically downregulated in AdipoRonPEG_5_ treated liver. D: Volcano plot comparing transcriptomes from AdipoRon-treated group versus AdipoRonPEG_5_-treated group. Metabolism related genes that is at least significantly changed (Log_10_ (*P* value) < −1.3) are annotated. The number of genes that were significantly downregulated (blue) or upregulated (red) are described at the bottom. E: Hierarchical clustering of genes involved in gluconeogenesis-related genes. F: Hierarchical clustering of genes related to AdipoR1 and AdipoR2 signaling pathway. G: The most upregulated genes by AdipoRon (left panel) and AdipoRonPEG5 (right panel) in the liver. H: The most downregulated genes by AdipoRon (left panel) and AdipoRonPEG5 (right panel) in the liver.

The robust impact on lowering ceramide species by AdipoRonPEG_5_ prompted us to analyze in greater detail the transcriptome profiles of liver tissues from HFD-fed mice treated with the drug. We performed hierarchical clustering of RNA-Seq data by grouping the genes according to their changes in response to the drug treatment (Fig. 6A). In reference to the gene expression levels in the drug-untreated mouse liver, we classified liver genes as downregulated (down), no change (nc), or upregulated (up) groups if their fold changes were lower than 0.9, between 0.9 and 1.1, or higher than 1.1 after drug treatments, respectively. According to the heatmap, we selected 3,522 genes for pathway analysis as Set 1 that were downregulated by adipoRonPEG_5_ treatment. Heatmaps of the transcriptome from AdipoRonPEG_5_ and AdipoRon-treated livers exhibit a distinct pattern of gene expression responses, despite both AdipoRon and AdipoRonPEG_5_ targeting adiponectin signaling (Fig. 6A). Additionally, principal component analysis shows that the liver transcriptome profiles are distinctly different among each group (Fig. 6B). In order to characterize the genes that were downregulated by AdipoRonPEG_5_ treatment, we performed GO pathway analysis with the Set 1 genes. GO analysis showed that triglyceride-metabolism-related pathways and glucose-metabolism-related pathways were enriched with Set 1 genes (Fig. 6C). In parallel with its effect on upregulating ceramidase-related genes (Fig. 5B), AdipoRonPEG_5_ downregulated triglyceride- and glucose-metabolism-related genes, including *stearoyl-CoA desaturase1* (*scd1*) that plays a pivotal role in lipogenesis and *pck1*, which encodes the rate-limiting enzyme for gluconeogenesis. The volcano plot comparing the transcriptomes between AdipoRon-treated versus AdipoRonPEG5-treated group further highlighted differentially expressed genes between these two groups (Fig. 6D), revealing AdipoRonPEG_5_ downregulating *fatty acid synthase* (*Fasn*) and stearoyl-CoA desaturase (*Scd1*), two genes that are vital for fatty acid synthesis. Among the genes that were upregulated by AdipoRonPEG5, acid ceramidase (*Asah1*) and uncoupling protein 2 (*Ucp2*) are known to be involved in the ceramide metabolism and in mediating the hepatoprotective activities of adiponectin (62) respectively (Fig. 6D). Given the substantial importance of the gluconeogenesis pathway in glucose homeostasis and its involvement in the glucose lowering effect of AdipoRonPEG_5_, we further examined the expression of genes involved in gluconeogenesis. Hierarchical clustering of gluconeogenesis-related genes clearly demonstrated the inhibitory effect of AdipoRonPEG_5_ on gluconeogenesis in the liver (Fig. 6E).

To further assess the mode of action of AdipoRonPEG_5_ with respect to the activation of adiponectin receptors and downstream signaling events, we extracted and analyzed the AdipoR1 and R2 signaling pathway genes. Interestingly, the effect of AdipoRonPEG_5_ suggested a preference toward amplifying AdipoR1 signaling pathway, while AdipoRon's effect is sided with AdipoR2 signaling (Fig. 6F). This echoes results reported in a previous study (27) proposing that increased insulin sensitivity upon AdipoRon treatment can restore diabetes-induced decrease in renal and cardiac AdipoR1 and AdipoR2 expression to the levels present in control db/m mice.

Next, we ranked the top 30 genes that went up (Fig. 6G) or down (Fig. 6H) after AdipoRon or AdipoRonPEG_5_ treatment. While AdipoRon highly upregulated genes involved in lipogenesis such as *ATP citrate lyase* (*Acly*) and *Scd1* (Fig. 6G, left panel), AdipoRonPEG_5_ induced prominent increases of *glutathione S-transferases* (*Gstm1 and Gstm2*) (Fig. 6G, right panel). On the other hand, the common genes by AdipoRon and AdipoRonPEG_5_ treatment include transcription factors such as *B*-*cell lymphoma6* (*Bcl6*) and *nuclear receptor subfamily1 groupD member1* (*Nr1d1*) (Fig. 6H). Downregulated genes by AdipoRonPEG5 are composed of gluconeogenesis and lipogenesis-related genes such as *Pck1*, *Scd1*, and *Fasn* (Fig. 6H, right panel).

## Discussion

AdipoRon is the first synthetic orally active AdipoR agonist (23) that was shown to exert actions similar to those of adiponectin, an antidiabetic and antiatherogenic adipokine (35, 63) resulting in enhanced insulin sensitivity and glucose tolerance in mice exposed to HFD. In addition to lowering serum glucose, injection of AdipoRon into leptin-deficient *ob*/*ob* mice results in reduced hepatic ceramide content, suggesting similar metabolic beneficial effects to those of adiponectin (64).

Despite its beneficial effects in mouse models (65, 66, 67, 68) AdipoRon's therapeutic potential is hampered by its low aqueous solubility (9 μg/ml in water, Fig. 1C). Even in a neutral phosphate buffer containing >30% DMSO, AdipoRon is only soluble to 0.25 mg/ml (https://www.caymanchem.com/pdfs/15941.pdf), limiting its treatment to oral administration at 50 mg kg^−1^ body weight over a period of 10–28 days, with a reported maximal plasma concentration of 11.8 μM (23). Since its introduction in 2013, there have been few attempts to optimize AdipoRon structure to improve its bioavailability. To further unlock adiponectin's therapeutic potential, it would be beneficial to engineer small molecules with improved bioavailability. This depends on several factors, the most important of which is solubility in an aqueous environment (69). Indeed, enhancing the solubility of lipophilic drugs may allow for more efficient routes of administration that bypass the need for solute absorption (70). Among the several strategies devised to enhance the solubility of lipophilic small molecules is the covalent modification with PEG or PEGylation. PEGylation offers other advantages, the chief among which is providing a nontoxic, nonimmunogenic handle for crafting novel bioconjugates for enhancing probe targetability (40, 71). Taking this into account, we designed several AdipoRon analogs having -OH or PEG groups at the para-position of the benzyl amine moiety to create PEG linkers that can be further functionalized to anchor targeting moieties for future studies. Initially, we assessed our design modification by a cell survival assay performed on INS-1 β-cells stressed with PA. Such an assay was based on several reports showing that adiponectin promotes hepatocytes, cardiomyocytes, and β-cell survival and function, in part through adiponectin-receptor-mediated ceramidase activity (12, 36) that reduces cellular ceramides and dihydroceramides. Ceramides and dihydroceramides are important classes of bioactive lipids that have been shown to correlate with a diverse array of metabolic diseases (72). These two lipids also serve as intermediates for the formation of the phosphorylated sphingoid base S1P (Fig. 3A), a potent inducer of proliferation and inhibitor of apoptosis (73). In addition to activating ceramidase, binding of adiponectin to AdipoR1 and AdipoR2 triggers the phosphorylation of AMPK and the inhibitory phosphorylation of its downstream target ACC, and the increased expression of *Pparα*, respectively (46, 74, 75, 76). Hepatic activation of AMPK and suppression of ACC, the rate-limiting enzyme in de novo lipogenesis, are associated with reduced lipogenesis and lipid accumulation (77).

Our initial assessment suggests that AdipoRon, in the presence of the nonionic surfactant Pluronic® F-127, can attenuate PA-induced cytotoxicity of INS-1 pancreatic β-cells in a dose-dependent manner. More importantly, modifications to the phenyl group of the benzylamine moiety in AdipoRon, which yielded AdipoRon-OH, AdipoRonPEG_2_Cl, AdipoRonPEG_5_, and AdipoRonPEG_6_HTL, suggest that the β-cell protective effects of AdipoRon can be enhanced as a result of PEG-mediated improvement in aqueous solubility (Fig. 2A). Concomitantly, mass spectrometric analysis of the ceramide levels in INS-1 cells showed that AdipoRonPEG_5_ treatment resulted in a reduction of C22:0 and C24:0 ceramides, which are particularly harmful for β-cell function and viability (78). In comparison with AdipoRon, AdipoRonPEG_5_ treatment resulted in a significantly greater reduction of C24:0 ceramide levels in pancreata of HFD-fed mice (Fig. 3).

Previous studies on sphingolipid contributions to metabolic dysfunction showed that the depletion of ceramides, particularly C16:0 and C18:0 ceramides, can prevent or even reverse the development of hepatic steatosis, as well as improve systemic glucose tolerance and insulin sensitivity in adult mice with HFD-induced obesity (74, 78). We also showed that circulating ceramide species, whose levels correlate with the severity of insulin resistance, reflect the degree of their breakdown in the liver and adipose tissue. With that in mind, we examined the effect of AdipoRon and AdipoRonPEG_5_ on ceramide levels in livers, pancreas, and subcutaneous adipose tissues of HFD-fed mice, as well as the levels of serum ceramide after 5 days of drug treatment. Unlike AdipoRon, AdipoRonPEG_5_-treated hepatic dihydroceramides were drastically reduced in the case of dC22:0, dC24:0, and dC24:1. More importantly, AdipoRonPEG_5_-treated hepatic sphingosine and S1P were markedly increased in comparison to AdipoRon. These findings also reflect the sharp decline in the serum ceramides levels, which was more pronounced with AdipoRonPEG_5_ versus AdipoRon treatment. Collectively, these data suggest that AdipoRonPEG_5_ is more effective in reducing lipotoxicity in the pancreas and liver of HFD-fed mice and is consistent with adiponectin cytoprotective effect.

We previously reported that adiponectin-mediated ceramide catabolism in pancreatic β-cells and cardiomyocytes is independent of AMPK activation (12). Our current findings suggest a similar effect from AdipoRonPEG5: while p-AMPK levels are higher in the liver of HFD-induced obese mice treated with AdipoRon than those treated with AdipoRonPEG_5_ (Fig. 5A), ceramide levels were reduced more pronouncedly by AdipoRonPEG5, accompanied by the formation of its antiapoptotic metabolite, sphingosine, and S1P (Fig. 5C–L).

In keeping with an expanding body of evidence that adiponectin (79) and AdipoRon (23, 64, 80) ameliorate insulin resistance and glucose intolerance via AdipoR activation, we investigated the ability AdipoRonPEG_5_ to recapitulate these effects and provide significant metabolic improvements. Indeed, STZ-treated adiponectin null mice had markedly improved glucose tolerance during an OGTT compared with littermates not treated with AdipoRonPEG_5_. This can be a direct effect of gluconeogenesis, the main determinant of serum glucose level under STZ-mediated insulinopenic condition (81). In a previous report, we found that adiponectin is critical for insulin-mediated suppression of lipolysis under insulinopenic conditions and with a low dose of insulin stimulation. However, AdipoRonPEG_5_ had nominal effect on triglyceride clearance. This suggests that even though AdipoRonPEG_5_ may potentiate insulin sensitivity, basal insulin levels may not be sufficient to significantly suppress lipolysis.

**Fig. 7:**
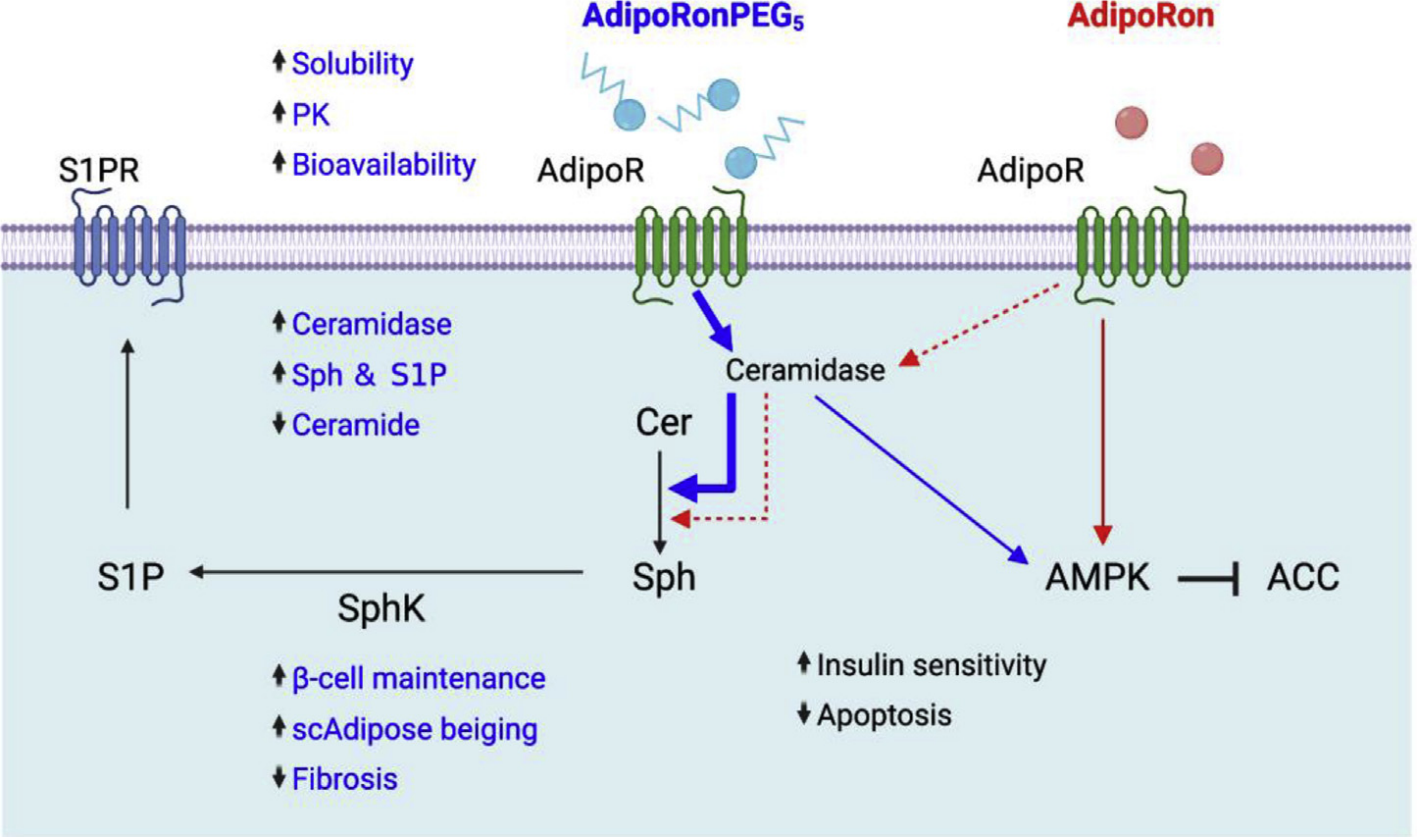
Pharmacological improvements of AdipoRonPEG_5_ enhance its action through AdipoR to exert pleiotropic benefits on metabolism, cell function, and survival. The cellular signaling and physiological outcomes of AdipoRonPEG_5_ and AdipoRon are shown in blue and red, respectively. Signaling pathways or biological functions common to both drugs are shown in black. The scheme mainly includes results from the current study for clarity.

In T2D mice, AdipoRonPEG_5_ positively affects glucose homeostasis as indicated by the improved insulin sensitivity. Along with the favorable effect on the hepatic Cer:S1P ratio, systemic test results suggest that the improvement of hepatic gluconeogenesis and hepatic insulin sensitivity mitigated the glucose level under HFD conditions. Based on our findings, we propose that, in vivo, AdipoRonPEG_5_ exerts its agonistic effects primarily by interacting with AdipoR in the liver. Mechanistically, AdipoRonPEG_5_ stimulates the cellular signaling cascades largely through ceramidase and, by doing so, subsequent AMPK activation (Fig. 7). In addition to promoting the intrinsic ceramidase activity of AdipoR (36) AdipoRonPEG_5_ also upregulated the expression of several ceramidase genes including alkaline ceramidases *Acer2* and *Acer3*; acid ceramidase *Asah1*; and neutral ceramidase *Asah2* (Fig. 5B). Consistently, a direct biochemical assay of ceramidase activity in the INS-1 cell lysate confirmed that AdipoRonPEG_5_ robustly reduced the level of deuterated ceramide substrates compared with the control or with AdipoRon (supplemental Fig. S7). The exact mode of action of AdipoRonPEG_5_ in upregulating ceramidase expression remains an interesting topic for the future investigation. Regardless, the ability of AdipoRonPEG_5_ in activating AdipoR and its potency in stimulating ceramidase both in vitro and in vivo are unprecedented and represent a major stride forward in engineering pharmacological agents to study adiponectin signaling and function.

As with all small molecules that serve as agonists in vivo, we cannot formally exclude the possibility that AdipoRonPEG_5_ and other AdipoRon derivatives act through additional receptors beyond adipoR1 and adipoR2. We rely very much on the original studies describing the dependence of AdipoRon on the AdipoRs. Nevertheless, we find the effects achieved by the new AdipoRon derivatives to be quite outstanding, and the molecules here certainly serve as a starting point for the development of even better ligands with improved target specificity and PK values in order to tap into the therapeutic potential of adiponectin for combating diabetes and metabolic diseases.

## Data availability

All data generated or analyzed during this study are included in this published article.

## Supplemental data

This article contains supplemental data.

## Conflict of interest

The authors declare that they have no conflicts of interest with the contents of this article.
