# Supplementary material for: PEGylated AdipoRon derivatives improve glucose and lipid metabolism under insulinopenic and high-fat diet conditions
Source: J Lipid Res. 2021 Jun 30;62:100095. doi: 10.1016/j.jlr.2021.100095 (PMC8327158; doi:10.1016/j.jlr.2021.100095)
Supplement: Supplementary file 1 — Supplemental data [file mmc1.docx]

# **SUPPLEMENTARY INFORMATION**

# **PEGylated AdipoRon Analogs Improve Glucose Homeostasis and Lipid Metabolism in Mice under Insulinopenic and High Fat Diet Conditions**

Toshiharu Onodera,^a,#^ Ebrahim Ghazvini Zadeh,^b,#^ Peng Xu,^c^ Ruth Gordillo,^b^ Zheng Guo,^c^ Nolwenn Joffin,^a^ Biao Yu,^c^ Philipp E. Scherer,^a,b,*^ Wen-hong Li ^b,d*^

*^a^* Touchstone Diabetes Center, Department of Internal Medicine, The University of Texas Southwestern Medical Center, Dallas, Texas

*^b^* 75390Department of Cell Biology, The University of Texas Southwestern Medical Center, Dallas, Texas, 75390

*^c^* State Key Laboratory of Bio-organic and Natural Products Chemistry, Shanghai Institute of Organic Chemistry, Chinese Academy of Sciences, Shanghai 200032

*^d^* Department of Biochemistry, The University of Texas Southwestern Medical Center, Dallas, Texas, 75390

# Equal Contribution

* Philipp.Scherer@UTSouthwestern.edu

* Wen-hong.li@utsouthwestern.edu

***List of Supplement Materials***

1. Syntheses and Characterization of AdipoRon analogues & Schemes 1-5;

2. Supplementary Figures S1 to S10;

3. Tables S1-8.

***1. Syntheses and Characterization of AdipoRon analogues & Schemes 1-5***

All reactions were carried out under nitrogen or argon with anhydrous solvents in flame-dried glassware, unless otherwise noted. Solvents were dried using a solvent purification system and used directly without further drying. The reagent-grade chemicals were used as supplied, except where noted. Analytical thin-layer chromatography was performed using silica gel 60 F254 glass plates. Compound spots were visualized by UV light (254 nm) or by heating with a solution with 10% H_2_SO_4_ in ethanol. Flash column chromatography was performed on silica gel. ^1^H NMR spectra were referenced using Me_4_Si (0 ppm), residual CHCl_3_ (^1^H NMR *δ* = 7.26 ppm, ^13^C NMR *δ* = 77.16 ppm), CD_3_OD (^1^H NMR *δ* = 3.31 ppm, ^13^C NMR *δ* = 49.00 ppm), D_2_O (^1^H NMR *δ* = 4.79 ppm). Peak and coupling constant assignments are based on ^1^H NMR, ^1^H–^1^H COSY, and ^1^H–^13^C HMQC experiments. Splitting patterns are indicated as s (singlet), d (doublet), t (triplet), q (quartet), and brs (broad singlet) for ^1^H NMR data. ESI-MS and MALDI-MS were run on an IonSpec Ultra instrument using HP5989A or VG Quattro MS.

Synthesis of **AdipoRonOH** is described in **Scheme 1**. Briefly, 4-hydroxybenxophenone **1** was alkylated with excess methyl bromoacetate in boiling acetone and the presence of K_2_CO_3_ to afford the methyl ester intermediate **2**. Hydrolysis to the corresponding acid **3** was achieved using aqueous NaOH, followed by treatment with SOCl_2_ and 1*H*-benzo[*d*][1,2,3]triazole to furnish **4**. The 1*H*-benzo[*d*][1,2,3]triazole moiety was substituted with the HCl salt of intermediate **6**, hence furnishing **AdipoRonOH**. The latter was used for further functionalization as shown in **Scheme 2**.

**Scheme 1** Synthesis of **AdipoRonOH**. *Reagents and conditions*: (a) methyl bromoacetate, K_2_CO_3_, acetone, Δ, 18 h, 90%; (b) NaOH, THF: H_2_O, Δ, 1 h, 99%; (c) 1*H*-benzo[*d*][1,2,3]triazole, SOCl_2_, Et_3_N, THF, rt, 3.5 h, 77%; (d) NaBH(OAc)_3_, 4-hydroxybenzaldehyde, CH_2_Cl_2_, rt, 12 h, 99%; (e) HCl, 1,4-dioxane, rt, 1 h, 99%; (f) **4**, Et_3_N, CH_3_CN, rt, 4 h, 59%.

**Methyl 2-(4-benzoylphenoxy)acetate (2)**: The title product was prepared according to the literature procedure^23^ with minor modification. Methyl bromoacetate (4.50 mL, 45.5 mmol, 1.8 equiv) was added to a suspension of 4-hydroxyphenyl)(phenyl)methanone **1** (5.00 g, 25.2 mmol, 1.0 equiv) and dry K_2_CO_3_ (6.96 g, 50.4 mmol, 2.0 equiv) in dry acetone (25 mL) under argon atmosphere. After stirring for 18 h with heating under reflux, the reaction mixture was filtered and the precipitate was concentrated under reduced pressure. The residue pale yellow solid was solubilized in methylene chloride (20 mL) and were poured onto cold hexanes. The resulting precipitate was collected by filtration, and was washed with hexanes to afford the title product as a beige solid (6.10 g, 90%). ^1^H NMR (CDCl_3_, 400 MHz): δ 7.86 – 7.77 (m, 2H), 7.79 – 7.70 (m, 2H), 7.60 – 7.51 (m, 1H), 7.46 (dd, *J* = 8.3, 7.0 Hz, 2H), 6.96 (d, *J* = 8.8 Hz, 2H), 4.71 (s, 2H), 3.82 (s, 3H).

**2-(4-Benzoylphenoxy)acetic acid (3)**: An aqueous solution of NaOH (20 mL, 1.5 M, 5.0 equiv) was added to a solution of **1** (2.70 g, 10.0 mmol) in THF:H2O (1:1, 40 mL), and the resulting biphasic solution was heated under reflux for 1 hour. After cooling to room temperature, HCl (1 N, 60 mL) was added slowly to the mixture at 0 ⁰C. The resulting precipitate was collected by filtration, and was washed with water and air dried. The resulting white solid was suspended in acetone (50 mL) and evaporated to afford the title product as a white solid (3.10, 99%).

**1-(1*H*-Benzo[*d*][1,2,3]triazol-1-yl)-2-(4-benzoylphenoxy)ethan-1-one (4)**: The title product was prepared according to the procedure described in *Tetrahedron Lett.*, **2013**, *54*, 5467–5469 with minor modification. SOCl_2_ (0.92 mL, 1.1 equiv, 12.0 mmol) was added to a solution of 1*H*-benzo[*d*][1,2,3]triazole (3.90 g, 32.8 mmol) in anhydrous THF (10 mL), and the resulting solution was stirred at room temperature for 20 min. To this solution was added dropwise a stirred solution of **3** (2.80 g, 10.9 mmol) and Et_3_N (1.5 mL, 1.0 equiv) in THF (15 mL) at 0 ⁰C. The reaction mixture was allowed to warm to room temperature and was stirred for 3 hours. The precipitate was filtered off and was washed with EtOAc. The solvent was evaporated and the resulting oil was dissolved in EtOAc (50 mL) and was washed with sat. Na_2_CO_3_ (3 x 100 mL) or until all the excess benzotriazole is eliminated. The organic layer was dried over MgSO_4_ and the solvent was evaporated under reduced pressure to afford the desired product as white solid (3.00 g, 77%). ^1^H NMR (CDCl_3_, 400 MHz) δ 8.28 (d, *J* = 8.2 Hz, 1H), 8.17 (d, *J* = 8.3 Hz, 1H), 7.86 (d, *J* = 8.8 Hz, 2H), 7.80 – 7.73 (m, 2H), 7.71 (t, *J* = 7.7 Hz, 1H), 7.57 (t, *J* = 7.6 Hz, 2H), 7.47 (t, *J* = 7.5 Hz, 2H), 7.12 (d, *J* = 8.7 Hz, 2H), 5.85 (s, 2H).

***tert*-Butyl (1-(4-hydroxybenzyl)piperidin-4-yl)carbamate (6)**: The title product was prepared according to the procedure described in *J. Med. Chem.*, **2002**, *45*, 654-662. A solution of *tert*-butyl piperidin-4-ylcarbamate **5** (2.00 g, 10.0 mmol) and 4-hydroxybenzaldehyde (1.34 g, 11.0 mmol) in CH_2_Cl_2_ (50 mL) was stirred at room temperature for 30 min. To the bright red solution was added NaBH(OAc)_3_ (4.24 g, 20.0 mmol) and the resulting solution was stirred overnight at room temperature. The reaction was quenched with H_2_O (5 mL), and the organic solvent was evaporated. EtOAc (50 mL) was then added and the organic layer was washed with sat. Na_2_CO_3_ (2 x 100 mL) and sat. NaCl (2 x 100 mL). The organic layer was then dried over MgSO_4_ and the solvent was evaporated to afford the title product as a colorless oil (3.00 g, 99%). ^1^H NMR (CDCl_3_, 400 MHz) δ 7.04 (d, *J* = 8.3 Hz, 2H), 6.62 (d, *J* = 8.1 Hz, 2H), 4.58 (d, *J* = 8.2 Hz, 1H), 3.40 (s, 3H), 2.92 – 2.74 (m, 2H), 2.08 (t, *J* = 11.7 Hz, 2H), 1.95 – 1.73 (m, 2H), 1.41 (s, 10H). HRMS (ESI) m/z calculated for C_17_H_27_N_2_O_3_^+^ [M+H]^+^: 307.2016, found 307.0652.

**4-Ammonio-1-(4-hydroxybenzyl)piperidin-1-ium chloride (7)**: HCl in 1,4-dioxane (4 N, 5 mL) was added to a solution of **6** (3.00 g, 10.0 mmol) in 1,4-dioxane (10 mL) at 0 ⁰C. The suspension was stirred at room temperature, and additional 1,4-dioxane (10 mL) was added due to the formation of precipitate. The solvent was evaporated after 1 hour, and the resulting solid was suspended in diethyl ether (50 mL) and the resulting solid was filtered off and dried under vacuum to afford the title compound as an off-white solid (2.72 g, 99%).

**AdipoRonOH**: A suspension of **4** (3.56 g, 10.0 mmol) and **7** (2.72, 10.0 mmol) in CH_3_CN (75 mL) was stirred at room temperature and was monitored by TLC (~ 4 hours). The solvent was evaporated under reduced pressure, and the resulting oil was suspended in EtOAc (50 mL) and was washed with sat. Na_2_CO_3_ (3 x 100 mL) and sat. NaCl (2 x 100 mL). The organic layer was dried over MgSO4 and the solvent was evaporated. The resulting crude oil was purified by column chromatography (2% → 10% MeOH in CH­_2_Cl_2_) to afford the title product as a white solid (2.50 g, 59%). ^1^H NMR (CDCl_3_, 400 MHz): δ 7.83 (d, *J* = 8.8 Hz, 2H), 7.74 (dd, *J* = 8.3, 1.4 Hz, 2H), 7.63 – 7.52 (m, 1H), 7.47 (t, *J* = 7.5 Hz, 2H), 7.08 (d, *J* = 8.4 Hz, 2H), 6.97 (d, *J* = 8.8 Hz, 2H), 6.67 (d, *J* = 8.4 Hz, 2H), 6.47 (d, *J* = 8.3 Hz, 1H), 4.54 (s, 2H), 4.02 – 3.82 (m, 1H), 3.43 (s, 2H), 2.97 – 2.75 (m, 2H), 2.23 – 2.05 (m, 2H), 2.01 – 1.83 (m, 2H), 1.66 – 1.47 (m, 2H). HRMS (ESI) m/z calculated for C_27_H_29_N_2_O_4_^+^ [M+H]^+^: 445.2122, found 455.0333.

**Scheme 2**. Derivatization of **AdipoRon-OH**.

**Scheme 3**. Synthesis of **AdipoRon-PEG_2_-Cl**. (a) TsCl, TEA, DMAP; (b) **AdipoRon-OH**, K_2_CO_3_, LiBr, 18-crown-6, MeCN, heat.

**General Procedure for the preparation of 8 and 10**

To a solution of *p*-tosylchloride (1.5 equiv.) in CH_2_Cl_2_ (0.2 M), alcohol substrate (1 equiv.) triethylamine (1.6 equiv.) and DMAP (0.1 equiv.) were added. Stirring at room temperature was continued until TLC indicated the consumption of starting material. The mixture was concentrated, and the residue was purified by column chromatography to yield the resulting tosylate.

**2-(2-Chloroethoxy)ethyltosylate (8):** 90%. ^1^H NMR (500 MHz, CDCl_3_) *δ* 7.80 (dd, *J* = 8.4, 1.9 Hz, 2H), 7.37 – 7.32 (m, 2H), 4.17 (qd, *J* = 3.7, 1.8 Hz, 2H), 3.69 (m, 4H), 3.54 (td, *J* = 5.8, 1.4 Hz, 2H), 2.45 (d, *J* = 2.9 Hz, 3H).

**AdipoRon-PEG_2_Cl**: To a solution of **8** (1.2 equiv.) and **AdipoRon-OH** (1 equiv.) in 5 mL acetonitrile, K_2_CO_3_ (2 equiv.), LiBr (1 equiv.) and 18-crown-6 (1 equiv.) were added, and the reaction was heated under reflux until TLC indicated the consumption of starting material (~ 36 h). The mixture was cooled to room temperature, filtered. The filtrate was concentrated in vacuo, and the residue was purified by silica gel chromatography (DCM/CH_3_OH = 20:1 to 15:1) to yield the desired product as a colorless oil (79%). ^1^H NMR (CDCl_3_, 500 MHz) *δ* 7.87 – 7.84 (m, 2H), 7.79 – 7.73 (m, 2H), 7.63 – 7.55 (m, 1H), 7.49 (t, *J* = 7.7 Hz, 2H), 7.24 (s, 2H), 7.03 – 6.97 (m, 2H), 6.91 – 6.85 (m, 2H), 6.41 (d, *J* = 8.3 Hz, 1H), 4.55 (s, 2H), 4.17 – 4.11 (m, 2H), 3.97 – 3.91 (m, 1H), 3.90 – 3.86 (m, 2H), 3.84 (t, *J* = 5.9 Hz, 2H), 3.66 (t, *J* = 5.9 Hz, 2H), 3.48 (s, 2H), 2.84 (s, 1H), 2.17 (s, 1H), 1.98 – 1.91 (m, 2H). ^13^C NMR (125 MHz, CDCl_3_) *δ* 195.3, 166.6, 160.4, 157.9, 137.9, 132.6, 132.2, 131.5, 130.4, 129.7, 128.3, 114.4, 114. 3, 71.5, 69.8, 67.5, 67.29, 62.20, 51.90, 46.23, 42.73, 31.87; ESI-MS *m/z* calcd for C_31_H_36_N_2_O_5_Cl [M+H]^+^ 551.2307, found 551.2315.

**Scheme 4**. Synthesis of **AdipoRon-PEG_5_-OH**. *Reagents and conditions*: (a) PMBOH, Amberlyst 15, Δ; (b) TsCl, TEA, DMAP; (c) AdipoRon-OH, K_2_CO_3_, LiBr, 18-crown-6, MeCN, reflux; (d) DDQ, DCM, pH = 7 buffer.

**2-(2-((4-Methoxybenzyl)oxy)ethoxy)ethan-1-ol (9):** To the solution of pentaethylene glycol in DCM (0.3 M), 4-methoxybenzyl alcohol (1 equiv.) and Amberlyst 15 (20 mg/mL) were added. Then the mixture was heated at 60 ^o^C for 4 hours. The resulting mixture was filtered, and the filtrate was concentrated in vacuo. Crude product was purified by flash chromatography to afford mono-protected glycol **9** in 53% yield. ^1^H NMR (CDCl_3_, 500 MHz) *δ* 7.29 – 7.23 (dd, *J* = 8.6, 1.5 Hz, 2H), 6.87 (dd, *J* = 8.6, 1.5 Hz, 2H), 4.49 (d, *J* = 1.4 Hz, 2H), 3.80 (s, *J* = 1.7 Hz, 3H), 3.73 – 3.57 (m, 20H).

***N*-{1-[4-(1-*p*-Methoxyphenyl)-3,6,9,12,15-pentaoxahexadecanylbenzyl]piperidin-4-yl}-2-(p-benzoylphenoxy)acetamide (11).** The title product was prepared similar to **AdipoRon-PEG_2_Cl** with a yield of 74%. ^1^H NMR (CDCl_3_, 500 MHz) *δ* 7.88 – 7.83 (m, 2H), 7.79 – 7.73 (m, 2H), 7.49 (t, *J* = 7.7 Hz, 2H), 7.26 (s, 2H), 7.23 (d, *J* = 8.1 Hz, 2H), 7.03 – 6.97 (m, 2H), 6.90 – 6.84 (m, 4H), 4.55 (s, 2H), 4.49 (s, 2H), 4.11 (dd, *J* = 5.8, 4.1 Hz, 2H), 3.85 (dd, *J* = 5.7, 4.2 Hz, 2H), 3.80 (s, 3H), 3.74 – 3.58 (m, 17H), 3.50 (s, 1H), 2.86 (s, 2H), 2.18 (s, 2H), 1.95 (d, *J* = 12.6 Hz, 2H), 1.69 (s, 2H).

**AdipoRon-PEG_5_-OH**. To a biphasic suspension of **11** (1 equiv.) in DCM: buffer pH 7 (v/v = 10: 1, 0.1 M), was added 2,3-dichloro-5,6-dicyano-1,4-benzoquinone, DDQ (5 equiv.), followed by vigorous stirring. Upon consumption of starting material as indicated by TLC, the reaction mixture was diluted with DCM; the organic layer was washed with saturated NaHCO_3_ and brine, dried over Na_2_SO_4_, and filtered. The filtrate was concentrated *in vacuo*, and the residue was purified by silica gel column (DCM/CH_3_OH = 20:1) to give the desired product as a colorless oil (78%). ^1^H NMR (CDCl_3_, 500 MHz) *δ* 7.88 – 7.82 (m, 2H), 7.79 – 7.73 (m, 2H), 7.58 (d, *J* = 7.5 Hz, 1H), 7.48 (t, *J* = 7.7 Hz, 2H), 7.19 (d, *J* = 8.5 Hz, 2H), 6.99 (d, *J* = 8.8 Hz, 2H), 6.89 – 6.83 (m, 2H), 6.39 (d, *J* = 8.3 Hz, 1H), 4.54 (s, 2H), 4.15 – 4.09 (m, 2H), 3.85 (dd, *J* = 5.7, 4.1 Hz, 2H), 3.74 – 3.70 (m, 4H), 3.67 (dd, *J* = 10.0, 4.7 Hz, 10H), 3.59 (dd, *J* = 5.4, 3.8 Hz, 2H), 3.42 (s, 2H), 2.79 (d, *J* = 11.6 Hz, 2H), 2.11 (s, 2H), 1.92 (dd, *J* = 9.1, 4.2 Hz, 2H), 1.51 (qd, *J* = 11.2, 3.8 Hz, 2H). ^13^C NMR (125 MHz, CDCl_3_) *δ* 195.31, 166.67, 160.38, 137.87, 132.64, 132.17, 131.58, 130.49, 129.75, 128.27, 114.44, 114.26, 72.56, 70.81, 70.58, 70.56, 70.54, 70.29, 69.71, 67.42, 67.27, 62.13, 61.69, 51.85, 31.72, 1.01; ESI-MS *m/z* calcd for C_37_H_49_N_2_O_9_ [M+H]^+^, 665.3433, found 665.3443.

**Scheme 5**. Synthesis of **AdipoRon-PEG_6_HTL**. *Reagents and conditions*: (a) PhI(OAc)_2_, TEMPO, CH_2_Cl_2_, H_2_O, rt; (b) Boc_2_O, CH_2_Cl_2_; (c) NaH, 1-chloro-6-iodohexane, 0 ⁰C → rt, THF, DMF; (d) TFA, DCM, 0 ⁰C → rt ; (e) **12**, CDI, Na_2_CO_3_, DMF, 50 ⁰C; (f) DDQ, CH_2_Cl_2_, H2); (g) TsCl, Et_3_N, DMAP; (h) **AdipoRon-OH**; Cs_2_CO_3_, DMF, 50 ⁰C.

**15-(4-Methoxyphenyl)-3,6,9,12,15-pentaoxahexadecanoic acid (12).** To a suspension of **9** (462 mg, 1.29 mmol) in DCM (6 mL) and H_2_O (3 mL), 2,2,6,6-tetramethylpiperidine-1-oxyl, TEMPO (61 mg, 0.39 mmol) and phenyliodine(III) diacetate (1.25 g, 3.87 mmol) were added, successively. Stirring was continued until TLC indicated disappearance of the starting material. The mixture was diluted with DCM; the organic layer was washed with saturated NaHCO_3_, and brine, dried over Na_2_SO_4_, and then concentrated was concentrated *in vacuo*. The resulting crude product was purified by column chromatography (DCM/MeOH = 5:1) to afford the desired product (277 mg, 0.75 mmol, 58%). ^1^H NMR (CDCl_3_, 500 MHz,) *δ* 7.30 (d, *J* = 6.3 Hz, 2H), 6.89 (d, *J* = 8.6 Hz, 2H), 4.52 (s, 2H), 4.15 (s, 2H), 3.82 (s, 3H), 3.77 – 3.74 (m, 2H), 3.69 (m, 12H), 3.64 – 3.61 (m, 2H); ESI-MS *m/z* calcd for C_18_H_28_NaO8^+^ [M+Na]^+^: 395.16; found: 395.2.

***tert*-Butyl [2-(2-hydroxyethoxy)ethyl]carbamate (13):** To a solution of 2-(2-aminoethoxy)ethanol (2 g, 19 mmol) in 50 mL of DCM, Boc_2_O (4.2 g, 19.0 mmol) was added, and stirring was continued for 2 h. The mixture was concentrated, and the residue was purified by column chromatography (PE/EA = 1:2) to afford the target product (3.8 g, 18.5 mmol, 97%). ESI-MS *m/z* calcd. for C_9_H_19_NNaO_4_^+^ [M+Na]^+^ 228.1; found: 228.1.

***tert*-Butyl [2-(6-Chlorohexyloxyethoxy)ethyl]carbamate (14).** Sodium hydride (580 mg, 14.4 mmol, 60% in mineral oil) was added portion-wise to a solution of **13** (2.433 g, 11.9 mmol) in THF (17 mL) and DMF (8.5 mL) at 0 ^⁰^C. After stirring for 30 min at this temperature, 1-chloro-6-iodohexane (4.0 g, 16.7 mmol) was added, and the reaction mixture was warmed to room temperature and stirred overnight. The reaction was quenched by water and was diluted with DCM. The organic layer was washed with brine, and dried over Na_2_SO_4_, and concentrated in vacuo. The residue was purified by column chromatography (PE: EA = 4: 1) to afford the target product **14** (1.76 g, 5.47 mmol, 46%). ^1^H NMR (CDCl3, 500 MHz) *δ* 3.61 (dd, *J* = 6.2, 3.5 Hz, 2H), 3.59 – 3.52 (m, 6H), 3.47 (t, *J* = 6.7 Hz, 2H), 3.32 (t, *J* = 5.0 Hz, 2H), 1.83 – 1.74 (m, 2H), 1.61 (q, *J* = 7.1 Hz, 2H), 1.45 (s, 11H), 1.42 – 1.35 (m, 2H).

**2-(6-Chlorohexyloxyethoxy)ethanamine (15).** Deprotection of **14** (1.74 g, 5.4 mmol) involved treatment with 4 mL of TFA in 10 mL DCM at 0 ⁰C for ~ 3 h. The mixture was diluted with ethyl acetate (50 mL), and the organic layer washed with saturated aqueous NaHCO_3_ (2 x 25 mL) and brine, dried over Na_2_SO_4_, evaporated to afford the target compound **15** (99%). ^1^H NMR (CDCl_3_, 500 MHz) *δ* 3.68 (t, *J* = 5.1 Hz, 2H), 3.62 (dd, *J* = 5.9, 3.2 Hz, 2H), 3.56 (dd, *J* = 5.9, 3.2 Hz, 2H), 3.51 (t, *J* = 6.7 Hz, 2H), 3.45 (t, *J* = 6.8 Hz, 2H), 3.12 (t, *J* = 5.1 Hz, 2H), 1.74 (q, *J* = 7.1 Hz, 2H), 1.56 (p, *J* = 7.0 Hz, 2H), 1.43 (p, *J* = 7.2 Hz, 2H), 1.37 – 1.27 (m, 2H).

***N*-[2-(6-Chlorohexyloxyethoxy)ethyl]-[15-(4-methoxyphenyl)]-3,6,9,12,15-pentaoxahexadecanamide acid (16).** A solution of 1,1’-carbonyldiimidazole (64 mg, 0.39 mmol) and **12** (122 mg, 0.33 mmol) in 2.5 mL anhydrous DMF was heated at 50 ^⁰^C for about 2 h. After cooling to rt, **15** (111 mg, 0.49 mmol) and sodium carbonate (87 mg, 0.83 mmol) were then added to the mixture, and stirring was continued for additional 8 h. The mixture was diluted with DCM, washed with 1 M HCl, 1 M NaOH and brine. The organic layer was dried over Na_2_SO_4_, and concentrated in vacuo, and the residue was purified by column chromatography (DCM/CH_3_OH = 15:1) to afford **16** (135 mg, 0.23 mmol, 71%). ^1^H NMR (CDCl_3_, 400 MHz,) *δ* 7.23 (d, *J* = 7.6 Hz, 2H), 7.20 – 7.09 (m, 1H), 6.84 (d, *J* = 8.1 Hz, 2H), 4.46 (s, 2H), 3.96 (s, 2H), 3.77 (s, 3H), 3.69 – 3.38 (m, 28H), 1.74 (p, *J* = 6.8 Hz, 2H), 1.57 (p, *J* = 7.0 Hz, 2H), 1.37 (dp, *J* = 31.0, 8.2 Hz, 4H). ^13^C NMR (CDCl_3_, 125 MHz) *δ* 169.99, 159.18, 130.29, 129.40, 113.75, 72.90, 71.27, 70.94, 70.67, 70.62, 70.60, 70.56, 70.30, 70.05, 69.82, 69.07, 55.29, 45.08, 38.59, 32.54, 29.48, 26.71, 25.43.

***N*-[2-(6-Chlorohexyloxyethoxy)ethyl]-3,6,9,12,15-pentaoxahexadecanamide (17).** To a suspension of **16** (135 mg, 0.23 mmol) in 0.3 mL DCM and 0.2 mL water, DDQ (261 mg, 1.15 mmol) was added and the mixture was stirred for ~ 30 min. The mixture was neutralized by aqueous NaHCO_3_, washed with brine, and dried over Na_2_SO_4_. The filtrate was concentrated in vacuo, and the residue was run through column chromatography (DCM/CH_3_OH = 10:1) to afford compound **17** (95 mg, 0.22 mmol, 94%). ^1^H NMR (CDCl_3_, 500 MHz) *δ* 4.01 (s, 2H), 3.76 – 3.41 (m, 28H), 1.81 – 1.73 (m, 2H), 1.63 – 1.55 (m, 2H), 1.48 – 1.41 (m, 2H), 1.37 (d, *J* = 6.9 Hz, 2H). ^13^C NMR (125 MHz, CDCl_3_) *δ* 169.0, 71.5, 70.2, 69.8, 69.6, 69.6, 69.5, 69.5, 69.3, 69.3, 69.3, 69.0, 68.8, 60.6, 44.1, 37.6, 31.5, 28.7, 28.4, 25.7, 24.4, 21.7, 13.1.

***N*-[2-(6-Chlorohexyloxyethoxy)ethyl]-15-tosyl-3,6,9,12,15-pentaoxahexadecanamide (18).** Yield: 81%. ^1^H NMR (500 MHz, CDCl_3_) *δ* 7.79 (d, *J* = 8.1 Hz, 2H), 7.34 (d, *J* = 8.0 Hz, 2H), 7.14 (t, *J* = 5.9 Hz, 1H), 4.14 (t, *J* = 4.8 Hz, 2H), 3.99 (s, 2H), 3.69 – 3.42 (m, 28H), 2.44 (s, 3H), 1.76 (q, *J* = 7.0 Hz, 2H), 1.62 – 1.55 (m, 2H), 1.48 – 1.41 (m, 2H), 1.39 – 1.33 (m, 2H). ^13^C NMR (125 MHz, CDCl_3_) *δ* 169.97, 144.84, 132.95, 129.84, 127.98, 71.26, 70.93, 70.77, 70.63, 70.59, 70.57, 70.52, 70.32, 70.30, 70.04, 69.81, 69.23, 68.71, 45.09, 38.58, 32.53, 29.48, 26.70, 25.43, 21.67.

**AdipoRon-PEG_6_HTL**. **AdipoRon-OH** (41 mg, 0.09 mmol) and **18** (68 mg, 0.11 mmol) was dissolved in 0.5 mL anhydrous DMF, then Cs_2_CO_3_ (75 mg, 0.23 mmol) was added. The mixture was heated to 50^o^C, and stirring was continued until TLC indicated disappearance of the starting materail (about 18 h). The reaction mixture was cooled down and diluted with DCM. The organic layer was washed with brine, dried over Na_2_SO_4_, filtered. The filtrate was concentrated, and the residue was purified by silica gel column (DCM/CH_3_OH = 10:1) to give compound the target compound (78.5 mg, 0.087 mmol, 97%). ^1^H NMR (500 MHz, CDCl_3_) *δ* 7.90 – 7.82 (m, 2H), 7.81 – 7.75 (m, 2H), 7.60 (t, *J* = 7.4 Hz, 1H), 7.50 (t, *J* = 7.6 Hz, 2H), 7.24 (d, *J* = 8.2 Hz, 2H), 7.06 – 6.98 (m, 2H), 6.92 – 6.85 (m, 2H), 6.50 (d, *J* = 8.4 Hz, 1H), 4.56 (s, 2H), 4.13 (t, *J* = 4.9 Hz, 2H), 4.01 (s, 2H), 3.98 – 3.91 (m, 1H), 3.87 (t, *J* = 4.9 Hz, 2H), 3.74 (dd, *J* = 6.1, 3.5 Hz, 2H), 3.73 – 3.65 (m, 10H), 3.65 – 3.43 (m, 14H), 2.93 (s, 2H), 2.22 (d, *J* = 12.4 Hz, 2H), 2.06 (d, *J* = 1.8 Hz, 1H), 1.96 (t, *J* = 6.8 Hz, 2H), 1.84 – 1.75 (m, 2H), 1.72 – 1.56 (m, 5H), 1.50 – 1.34 (m, 6H), 0.94 – 0.83 (m, 2H). ^13^C NMR (125 MHz, CDCl_3_) *δ* 195.4, 170.0, 166.8, 160.4, 137.9, 132.7, 132.2, 131.6, 130.7, 129.8, 128.3, 114.4, 114.3, 71.3, 70.9, 70.8, 70.6, 70.6, 70.6, 70.3, 70.0, 69.8, 69.7, 67.4, 67.2, 61.9, 51.7, 45.1, 38.6, 32.5, 31.9, 31.4, 30.3, 29.7, 29.7, 29.6, 29.5, 29.4, 26.7, 25.4, 22.7, 14.2; ESI-MS *m/z* calcd for C_47_H_67_N_3_O_7_Cl [M+H]^+^, 884.4459, found 884.4463.

**Figure S1.** Confocal microscopic analysis of palmitate-induced cell death in INS-1 β-cells stained with Hoechst 33342 and PI. INS-1 cells were treated for 24 h under various conditions including: (A) Serum-free RPMI containing 0.5% BSA; (B-G) Mediums containing palmitate (0.25 mM) in serum-free RPMI (B), RPMI with 10% FBS (C), serum-free RPMI with 10 nM Adiponectin (D), or with 10 µM AdipoRon (E), 10 µM AdipoRonPEG_5_ (F), or 10 µM AdipoRonPEG_6_HTL (G). Hoechst 33342 stains the condensed chromatin more brightly. PI stains dead cells. Scale bar = 10 µm.

**Figure S2.** Palmitate-induced cell death inhibition by selected AdipoRon analogs.

(A) Structures of AdipoRonPEG_12_ and AdipoRonPEG_12_HTL. (B, C) INS-1 beta cells were treated with incremental dosages of AdipoRonPEG_12_ (B) and AdipoRonPEG_12_HTL (C) for 24 h in the presence of palmitate (0.25 mM). The cells were then labeled with PI (1 mg/mL) for 5 min and measured on a fluorescence plate reader. PI fluorescence intensity was normalized against control cells that were not stressed with palmitate. Results are expressed as mean ± SEM (n = 3-6). *P < 0.05; **P < 0.01; ***P < 0.001, ****P < 0.0001 vs. 0 mM of compound.

**Figure S3.** Body weight reduction by AdipoRonPEG_6_HTL treatment.

Wild type mice were fed high fat diet for 4 weeks and treated with AdipoRonPEG_6_HTL (0.25, 2.5 or 5mg/kg) twice a day for 3 or 5 days. (A) Bodyweight was measured at day0 and day5 (n=5). (B) Bodyweight was measured at day0 and day3 (n=5). Data are mean ± SEM. **P* < 0.05

**Figure S4.** AdipoRonPEG_5_ does not alter genes that are involved in maintaining the functional state of pancreatic β-cells, but it upregulates thermogenesis-related genes in BAT. (**A**) Pancreatic gene expressions of insulin secretion related genes in AdipoRonPEG5 treated High fat diet fed mice (n=4 control n=5 AdipoRonPEG_5_). (**B**) Thermogenesis-related gene expression in brown adipose tissue (BAT) from 4 weeks HFD fed cohort (n = 5). Data are mean ± SEM. **P* < 0.05

**Figure S5.** AdipoRonPEG_5_ attenuates fibrosis in subcutaneous adipose tissue. (A and B) Wild type mice were fed high fat diet for 4 weeks or 7 months and treated with AdipoRonPEG_5_ (5 mg/kg) twice a day for 5 days. Subcutaneous adipose tissues were harvested for qPCR analysis. (A) Fibrosis related gene expression of subcutaneous adipose tissue from 4-weeks HFD fed cohort (n=5). (B) Fibrosis related gene expression of subcutaneous adipose tissue from 7-months HFD fed cohort (n=5). Data are mean ± SEM. **P* < 0.05

**Figure S6.** The effect of AdipoRonPEG_5_ (5 mg/kg) on gene expression in adipocytes after cold exposure (4 ℃). (A) Browning related gene expressions of subcutaneous white adipose tissue after cold exposure (n=6). (B) Brown adipose tissue marker expressions are quantified in brown adipose tissue after cold exposure (n=6).

**Figure S7. In vitro ceramidase activity assay**

INS-1 cells were treated with AdipoRon or AdipoRonPEG5 for 24 hours in the presence of palmitate. Labeled Ceramides were added to the cell lysate and incubated for 15 minutes. The relative abundance ((Metabolite peak Area / Internal standard Peak Area) / Total protein Content) were determined by mass spectrometry.

(A) Relative abundance of Ceramide 16:0–d7 (n=10) (B) Relative abundance of Ceramide 18:0–d7 (n=10) (C) Relative abundance of Ceramide 24:0–d7 (n=10) (D) Relative abundance of Ceramide 24:1–d7 (n=10) Data are mean ± SEM. **P* < 0.05 and ***P* < 0.01

**Figure S8. AdipoRonPEG_5_ induces the reduction of mouse pancreatic ceramide species levels.** Wild type mice were fed high fat diet for 4 weeks and treated with AdipoRon or AdipoRonPEG_5_ (5mg/kg) twice daily for 5 days before harvesting tissues. Pancreatic ceramide species levels were determined by mass spectrometry (n=5). Data are mean ± SEM. ***P* < 0.01.

**Figure S9.** Tissue ceramide level after AdipoRonPEG_6_HTL treatment. (A-F) Wild type mice were fed high fat diet for 4 weeks and treated with AdipoRonPEG_6_HTL (0.25 or 2.5mg/kg) twice a day for 5days followed by harvesting tissues. Ceramide species levels in the liver, subcutaneous and adipose tissue and pancreas were determined by mass spectrometry (n=5). Data are analyzed by two-way ANOVA with Tukey’s test. Data are mean ± SEM. **P* < 0.05, ***P* < 0.01, ****P* < 0.001 and *****P* < 0.0001

**Figure S10.** Tissue sphingoid bases level after AdipoRonPEG_6_HTL treatment. C57Bl/6 mice were fed high fat diet for 4 weeks and treated with AdipoRonPEG_5_HTL (0.25 or 2.5mg/kg) twice a day for 5 days followed by harvesting tissues. Sphingoid bases levels in the liver, subcutaneous and adipose tissue and pancreas were determined by mass spectrometry (n=5). Data are analyzed by two-way ANOVA with Tukey’s test. Data are mean ± SEM. **P* < 0.05, ***P* < 0.01, ****P* < 0.001 and *****P* < 0.0001.

**Table S1 to S8**

**Table S1. Genotyping Sequence for global adiponectin KO mice**

Primer Sequence

ApnWT F TTGGACCCCTGAACTTGCTTCACACC

ApnWT R TCCTGAGTTCAATTCCCAGCACCCAC

ApnKO R GGATGCGGTGGGCTCTATGGCTTC

WT: ApnWT F + WT R = 237 bp.

KO: ApnWT F + ApnKO R = 151 bp.

**Table S2. Primer information for qPCR**

Fibrosis

| Primer | Sequence |
| --- | --- |
| Collagen1a1 F | GCTCCTCTTAGGGGCCACT |
| Collagen1a1 R | CCACGTCTCACCATTGGGG |
| Collagen3a1 F | CTGGAGAACCTGGTGCAAAT |
| Collagen3a1 R | CCTCGGAAGCCACTAGGAC |
| Tgfb1 F | ACCATGCCAACTTCTGTCTG |
| Tgfb1 R | CGGGTTGTGTTGGTTGTAGA |
| Fn1 F | GCCCTGGTTTGTACCTGCTA |
| Fn1 R | GGAATCTTTAGGGCGCTCAT |
| Acta2 F | GTACCACCATGTACCCAGGC |
| Acta2 R | GCTGGAAGGTAGACAGCGAA |
| Timp1 F | CCCCAGAAATCAACGAGACCA |
| Timp1 R | ACTCTTCACTGCGGTTCTGG |

(continued)

| Pancreas (Table S2) |  |
| --- | --- |
| Primer | Sequence |
| Rxra F | ATGGACACCAAACATTTCCTGC |
| Rxra R | CCAGTGGAGAGCCGATTCC |
| Foxa2 F | GGAGGCAAGAAGACCGCTC |
| Foxa2 R | CCTTTAGCTCGCTTAGGCCAC |
| Creb1 F | AGCAGCTCATGCAACATCATC |
| Creb1 R | AGTCCTTACAGGAAGACTGAACT |
| Vamp2 F | GCTGGATGACCGTGCAGAT |
| Vam2 R | GATGGCGCAGATCACTCCC |
| Sur1 F | GAGATCGCTGAGGGTATCCTG |
| Sur1 F | TTGGGGAAGTTAGAGGTCTCAAT |
| Serca2 F | GAGAACGCTCACACAAAGACC |
| Serca2 R | CAATTCGTTGGAGCCCCAT |
| Serca3 F | CGTCGCTTCTCGGTGACAG |
| Serca3 R | AAGAGGTCCTCAAACTGCTCC |
| Glp1r F | ACGGTGTCCCTCTCAGAGAC |
| Glp1r R | ATCAAAGGTCCGGTTGCAGAA |
| Ins1 F | TGGTCCCCACCTGGTAAAG |
| Ins1 R | CACTTGTGGGTCCTCCACTT |
| Ins2 F | AGCCCTAAGTGATCCGCTACAA |
| Ins2 R | CATGTTGAAACAATAACCTGGAAGA |
| Mafa F | CCAGCTGGTATCCATGTCC |
| Mafa R | TTCTGTTTCAGTCGGATGACC |
| Nkx6.1 F | AAACACACCAGACCCACGTT |
| Nkx6.1 R | TCTCTCTGGTCCTGCCAAGT |
| Pdx1 F | CCTTTCCCGTGGATGAAAT |
| Pdx1 R | ACGGGTCCTCTTGTTTTCCT |
| Ngn3 F | AGTGCTCAGTTCCAATTCCAC |
| Ngn3 R | CGGCTTCTTCGCTTTTTGCTG |
| Neurod1 F | ATGACCAAATCATACAGCGAGAG |
| Neurod1 R | TCTGCCTCGTGTTCCTCGT |
| Irs1 F | GGGGGTTTGGAGAAGAGTCTTAACTACATAG |
| Irs1 R | GGTCATTTAGGTCTTCATTCTGCTGTG |
| Irs2 F | GGAGCCGGACCCGTAGCC |
| Irs2 R | TGGTAGCGCTTCACTCTTTCACG |
| Pcsk1 F | GACCTGCACAATGACTGCAC |
| Pcsk1 R | GGTCCAGACAACCAGATGCT |
| Pcsk2 F | GGCGTGTTTGCATTAGCTTT |
| Pcsk2 R | GCACAGTCAGATGTTGCATGT |

**Table S3. Western blot antibody information**

**Table S4.** Summary of the ceramide levels in INS1 cells (pg/mg) upon AdipoRonPEG_5_ treatment..

| **Species** | | **Control** | | | **AdipoRonPEG_5_ (1 μM)** | | | **AdipoRonPEG_5_ (20 μM)** | | |
| --- | --- | --- | --- | --- | --- | --- | --- | --- | --- | --- |
| Ceramides | 14:0 | 6.08 | 9.35 | 10.90 | 11.37 | 8.34 | 8.44 | 7.61 | 10.59 | 13.47 |
|  | 16:0 | 52.31 | 121.59 | 83.09 | 132.31 | 60.25 | 97.74 | 93.09 | 72.49 | 264.29 |
|  | 18:0 | 107.08 | 231.69 | 185.27 | 285.84 | 108.83 | 172.70 | 170.74 | 152.01 | 442.08 |
|  | 18:1 | 1.39 | 2.84 | 2.14 | 2.60 | 1.60 | 2.34 | 2.21 | 1.71 | 3.76 |
|  | 20:0 | 101.62 | 125.52 | 125.31 | 81.47 | 94.91 | 71.51 | 75.99 | 78.87 | 86.19 |
|  | 22:0 | 579.38 | 688.51 | 689.16 | 502.13 | 501.01 | 352.24 | 441.95 | 372.15 | 345.37 |
|  | 24:0 | 1099.71 | 1157.84 | 1271.82 | 985.21 | 942.22 | 715.38 | 951.19 | 835.47 | 827.24 |
|  | 24:1 | 443.92 | 522.80 | 527.60 | 415.25 | 405.60 | 306.31 | 400.62 | 388.65 | 377.12 |
|  | 26:0 | 13.69 | 16.17 | 17.33 | 13.63 | 11.39 | 10.09 | 10.66 | 9.87 | 9.40 |
|  | 26:1 | 4.85 | 6.05 | 6.09 | 4.83 | 4.31 | 3.39 | 4.43 | 3.78 | 3.59 |
| Dihydroceramides | 16:0 | 13.88 | 23.48 | 17.15 | 20.35 | 15.35 | 22.55 | 12.98 | 10.56 | 24.81 |
|  | 18:0 | 52.80 | 63.63 | 84.58 | 114.43 | 74.94 | 73.63 | 32.83 | 48.23 | 94.22 |
|  | 20:0 | 35.98 | 31.75 | 39.94 | 37.94 | 40.41 | 33.11 | 15.72 | 18.47 | 22.59 |
|  | 22:0 | 104.00 | 103.22 | 120.38 | 124.23 | 124.34 | 96.61 | 46.89 | 48.41 | 48.91 |
|  | 24:0 | 115.02 | 106.56 | 135.34 | 149.67 | 168.38 | 120.04 | 62.29 | 62.49 | 58.93 |
|  | 24:1 | 40.77 | 40.95 | 45.05 | 47.95 | 51.21 | 34.41 | 23.79 | 23.03 | 21.44 |

| **Tissue** | **Sample Name** | **Sphingosine** | **Sphinganine** | **Deoxysphingosine** | **Sphingosine1P** | **DeoxySphinganie** | **Sphingaine1P** |
| --- | --- | --- | --- | --- | --- | --- | --- |
| Liver | Control | 1119426 ± 157137.39 | 418922.9 ± 51074.29 | 479.5 ± 94.13 | 240847.8 ± 59008.58 | 18057.5 ± 5444.41 | 85494.5 ± 16727.93 |
|  | AdipoRon | 1192462 ± 75216.97 | 299552.9 ± 52030.89 | 554.4 ± 112.17 | 178631.8 ± 67607.36 | 16855.9 ± 5197.82 | 55129.9 ± 14495.18 |
|  | AdipoRonPEG_5_ | 1814140 ± 638775.97 | 567456.6 ± 215845.28 | 674.4 ± 179.33 | 423169.5 ± 201268.1 | 17056.5 ± 5841.85 | 124644.1 ± 37940.82 |
| Pancreas | Control | 211 ± 16.13 | 23.3 ± 4.52 | 0.0 ± 0.0 | 83.3 ± 17.8 | 2.1 ± 0.54 | 11.8 ± 11.15 |
|  | AdipoRon | 217.3 ± 27.75 | 25.9 ± 3.48 | 0.0 ± 0.0 | 90.3 ± 22.26 | 2.1 ± 0.29 | 17.5 ± 11.69 |
|  | AdipoRonPEG_5_ | 219.2 ± 31.51 | 34.5 ± 9.74 | 0.0 ± 0.0 | 113.2 ± 32.02 | 2.4 ± 0.49 | 25.6 ± 5.99 |
| scWAT | Control | 64477.7 ± 40013.41 | 34867.3 ± 18485.78 | 0.0 ± 0.0 | 45387.7 ± 21079.21 | 1451.5 ± 952.88 | 42180.2 ± 15546.49 |
|  | AdipoRon | 49182.2 ± 32181.22 | 29337.6 ± 16700.68 | 0.0 ± 0.0 | 37612.5 ± 17941.43 | 1039.3 ± 1085.07 | 35443.6 ± 15686.23 |
|  | AdipoRonPEG_5_ | 65862.8 ± 45362.44 | 35831.5 ± 25048.56 | 0.0 ± 0.0 | 45575.1 ± 20925.35 | 1329.3 ± 1128.06 | 40997.6 ± 18111.26 |
| Serum | Control at day 0 | 39067.1 ± 6599.4 | 39196.3 ± 11343.48 | 0.0 ± 0.0 | 796722.5 ± 76593.09 | 0.0 ± 0.0 | 276790.7 ± 47433.36 |
|  | Control at day 5 | 29350.2 ± 17685.97 | 24522.8 ± 20234.78 | 0.0 ± 0.0 | 1022213.2 ± 140527.59 | 0.0 ± 0.0 | 459650.7 ± 69103.81 |
|  | AdipoRon at day 0 | 43193.4 ± 8183.0 | 45226.5 ± 8665.07 | 0.0 ± 0.0 | 774207.2 ± 25661.64 | 0.0 ± 0.0 | 250934.3 ± 36218.42 |
|  | AdipoRon at day 5 | 18235.7 ± 1946.06 | 11725.4 ± 1939.02 | 0.0 ± 0.0 | 1139765.2 ± 69403.83 | 0.0 ± 0.0 | 423112.6 ± 48511.44 |
|  | AdipoRonPEG_5_ at day 0 | 34674.5 ± 9280.02 | 40000.3 ± 8021.4 | 0.0 ± 0.0 | 626634.8 ± 40618.85 | 0.0 ± 0.0 | 236098.7 ± 23069.11 |
|  | AdipoRonPEG_5_ at day 5 | 21363.8 ± 828.59 | 15276.5 ± 1962.03 | 0.0 ± 0.0 | 1121550.6 ± 208889.89 | 0.0 ± 0.0 | 512041.7 ± 138152.22 |

**Table S5**. Average (pg/mg) and standard deviation of sphingoid lipids in liver, pancreas, scWAT and serum of HFD-mice with and without (control) treatment of AdipoRon or AdipoRonPEG_5_.

| **Tissue**  **Table S6**. Average (pg/mg) and standard deviation of glycosphingolipids in liver, pancreas, scWAT and serum of HFD-mice with and without (control) treatment of AdipoRon and AdipoRonPEG_5_. | **Sample Name** | **Hexosyl 14:0** | **Lactosyl 16:0** | **Hexosyl 16:0** | **Lactosyl 18:0** | **Hexosyl 18:0** | **Hexosyl 20:0** | **Lactosyl 22:0** | **Lactosyl 24:1** | **Hexosyl**  **22:0** | **Hexosyl 24:1** | **Lactosyl C24:0** | **Hexosyl**  **24:0** |
| --- | --- | --- | --- | --- | --- | --- | --- | --- | --- | --- | --- | --- | --- |
| Liver | Control | 4811.6 ± 869.68 | 408426.2 ± 145968.0 | 1653305.4 ± 601624.66 | 0.0 ± 0.0 | 7489.4 ± 2700.59 | 101476.3 ± 27873.87 | 400239.0 ± 33856.72 | 291203.4 ± 44446.32 | 21225261.5 ± 3659120.73 | 5882876.7 ± 1654573.46 | 222009.1 ± 66015.57 | 8165783.3 ± 2251660.6 |
|  | AdipoRon | 6839.3 ± 2807.56 | 401293.8 ± 92547.01 | 2057918.6 ± 682419.73 | 0.0 ± 0.0 | 13702.3 ± 11183.27 | 139906.7 ± 74494.86 | 604972.0 ± 219908.19 | 424590.5 ± 191122.31 | 21992555.3 ± 7887275.74 | 7250991.4 ± 2638110.11 | 317251.2 ± 183613.54 | 9407290.2 ± 3691767.57 |
|  | AdipoRonPEG_5_ | 9405.4 ± 4098.51 | 857779.3 ± 221964.15 | 2563091.7 ± 636477.68 | 0.0 ± 0.0 | 8702.7 ± 4241.12 | 91341.0 ± 29325.4 | 403630.8 ± 109007.39 | 403865.6 ± 85387.31 | 18299628.4 ± 6151493.38 | 8806042.3 ± 2575774.26 | 284732.8 ± 96368.82 | 9345685.4 ± 2422218.78 |
| Pancreas | Control | 3.9 ± 1.64 | 918.6 ± 250.47 | 2748.4 ± 677.05 | 54.7 ± 3.58 | 6.9 ± 3.04 | 113.8 ± 43.25 | 194.6 ± 40.2 | 235.4 ± 37.55 | 6419.7 ± 720.48 | 1134.1 ± 605.75 | 1746.3 ± 298.72 | 3434.2 ± 256.04 |
|  | AdipoRon | 3.8 ± 0.85 | 975.8 ± 208.21 | 2561.5 ± 453.4 | 52.8 ± 9.76 | 7.3 ± 3.14 | 112.7 ± 34.01 | 223.0 ± 50.43 | 310.1 ± 93.68 | 6774.3 ± 637.6 | 1427.8 ± 124.45 | 2057.1 ± 422.83 | 3358.5 ± 311.97 |
|  | AdipoRonPEG_5_ | 4.2 ± 0.35 | 1043.4 ± 60.99 | 2792.5 ± 371.94 | 61.7 ± 15.72 | 8.8 ± 4.08 | 111.6 ± 39.83 | 258.7 ± 89.43 | 337.2 ± 90.18 | 5728.8 ± 1888.49 | 1360.1 ± 170.0 | 2121.1 ± 714.63 | 3233.9 ± 524.83 |
| scWAT | Control | 4932.3 ± 4026.86 | 39101.5 ± 40032.18 | 1797139.1 ± 1241823.49 | 0.0 ± 0.0 | 1032674.6 ± 607888.8 | 640424.1 ± 329524.36 | 38427.4 ± 26602.0 | 73307.8 ± 35349.12 | 6336413.4 ± 3686150.43 | 12921352.2 ± 8140289.71 | 40348.3 ± 33179.08 | 24962024.2 ± 18389080.86 |
|  | AdipoRon | 3863.9 ± 2313.65 | 45792.6 ± 45798.33 | 1558779.8 ± 419292.53 | 0.0 ± 0.0 | 879349.9 ± 318302.5 | 615610.3 ± 235007.39 | 49816.7 ± 23898.61 | 86367.6 ± 22529.94 | 6375434.2 ± 2782528.88 | 11496554.3 ± 5647659.03 | 55460.5 ± 33843.98 | 21878746.0 ± 11579620.12 |
|  | AdipoRonPEG_5_ | 5193.4 ± 2870.39 | 44866.5 ± 53044.09 | 1715352.2 ± 544724.48 | 0.0 ± 0.0 | 988756.1 ± 523374.82 | 624871.6 ± 310850.97 | 41846.1 ± 19236.41 | 71016.8 ± 18649.91 | 5932684.2 ± 2348171.67 | 13838465.2 ± 7503242.47 | 41489.9 ± 21166.47 | 25627611.9 ± 14360009.57 |
| Serum | Control at day 0 | 3767.5 ± 1125.75 | 95989.6 ± 25493.79 | 3285836.9 ± 436908.15 | 0.0 ± 0.0 | 57438.5 ± 18206.84 | 231341.4 ± 100793.63 | 72605.2 ± 10926.4 | 98892.4 ± 17186.11 | 13135001.5 ± 3910986.66 | 3833506.1 ± 873634.03 | 115411.7 ± 19706.22 | 1477067.5 ± 398318.75 |
|  | Control at day 5 | 1883.7 ± 736.98 | 78171.2 ± 10397.5 | 3169163.4 ± 550863.3 | 0.0 ± 0.0 | 50481.2 ± 12116.01 | 191187.5 ± 40279.48 | 53187.6 ± 7668.25 | 102362.3 ± 7257.83 | 8257800.3 ± 1714866.73 | 2430173.8 ± 419462.66 | 116277.8 ± 6477.9 | 1147046.9 ± 324638.49 |
|  | AdipoRon at day 0 | 2960.2 ± 601.74 | 94073.6 ± 8945.71 | 3024488.2 ± 155463.13 | 0.0 ± 0.0 | 47892.4 ± 4333.97 | 182974.2 ± 30498.44 | 60785.8 ± 9718.01 | 85880.2 ± 22412.15 | 12192915.9 ± 1475247.57 | 3535698.6 ± 346272.3 | 103502.4 ± 17366.51 | 1341147.2 ± 57498.17 |
|  | AdipoRon at day 5 | 2960.2 ± 601.74 | 94265.2 ± 16983.56 | 3736352.2 ± 191696.45 | 0.0 ± 0.0 | 63748.7 ± 18002.6 | 245486.5 ± 74132.09 | 58253.0 ± 5503.82 | 124049.2 ± 19300.14 | 9390882.1 ± 688490.25 | 3036231.9 ± 367214.43 | 122340.7 ± 11760.71 | 1430423.2 ± 157556.06 |
|  | AdipoRonPEG_5_ at day 0 | 2960.2 ± 1473.95 | 97139.2 ± 3984.55 | 3186431.1 ± 124319.92 | 0.0 ± 0.0 | 50804.8 ± 12885.62 | 212177.0 ± 30736.45 | 67539.8 ± 8954.59 | 91085.1 ± 17618.54 | 12426084.7 ± 1134587.25 | 4176146.7 ± 370105.27 | 106966.9 ± 12842.2 | 1547284.6 ± 152581.98 |
|  | AdipoRonPEG_5_ at day 5 | 2152.8 ± 1534.14 | 90624.9 ± 13005.26 | 4458327.8 ± 279958.81 | 0.0 ± 0.0 | 102580.4 ± 20477.28 | 312105.6 ± 82139.38 | 65007.0 ± 14500.42 | 157880.8 ± 14580.32 | 7780658.1 ± 1388198.99 | 3454314.7 ± 273336.65 | 128620.1 ± 18235.69 | 1624021.9 ± 177684.63 |

**Table S7**. Average (pg/mg) and standard deviation of dihydroceramides in liver, pancreas, scWAT and serum of HFD-mice with and without (control) treatment of AdipoRon or AdipoRonPEG_5_.

| **Tissue** | **Sample Name** | **dCer 16:0** | **dCer 18:0** | **dCer 20:0** | **dCer 22:0** | **dCer 24:1** | **dCer 24:0** |
| --- | --- | --- | --- | --- | --- | --- | --- |
| Liver | Control | 11193.9 ± 8139.21 | 30198.7 ± 14092.11 | 22915.9 ± 16868.47 | 311037 ± 76435 | 114551.6 ± 4190.72 | 441827.7 ± 59973.99 |
|  | AdipoRon | 18233.3 ± 21176.21 | 45901.3 ± 42922.13 | 37867.7 ± 27772.5 | 385034 ± 150652 | 130075.8 ± 53105.06 | 364847.1 ± 73663.91 |
|  | AdipoRonPEG_5_ | 7004.1 ± 1704.17 | 30523.3 ± 10512.82 | 17088.9 ± 5527.47 | 85360 ± 20574 | 64056.2 ± 12063.67 | 355838.6 ± 67813.11 |
| Pancreas | Control | 3.5 ± 1.12 | 0.0 ± 0.0 | 1.4 ± 0.32 | 25.1 ± 15.04 | 23.1 ± 9.48 | 198.1 ± 49.08 |
|  | AdipoRon | 3.8 ± 1.52 | 0.0 ± 0.0 | 2.0 ± 0.18 | 24.6 ± 9.85 | 24.0 ± 10.28 | 206.7 ± 49.68 |
|  | AdipoRonPEG_5_ | 5.7 ± 3.12 | 0.0 ± 0.0 | 1.9 ± 0.7 | 54.2 ± 43.08 | 47.3 ± 31.67 | 302.0 ± 155.43 |
| scWAT | Control | 14626.8 ± 3786.22 | 19797.3 ± 3513.43 | 74604.0 ± 44181.69 | 96183 ± 6549.33 | 113262.1 ± 27790.63 | 116522.1 ± 29114.01 |
|  | AdipoRon | 14580.7 ± 4393.04 | 17597.9 ± 5214.49 | 80869.1 ± 48824.3 | 84488 ± 14825 | 93338.7 ± 29289.58 | 96632.5 ± 27938.2 |
|  | AdipoRonPEG_5_ | 12585.7 ± 3750.43 | 16110.9 ± 4383.33 | 82646.0 ± 51960.31 | 65558.1 ± 8753.52 | 103545.6 ± 21754.88 | 87756.9 ± 10704.97 |
| Serum | Control at day 0 | 15717.6 ± 5422.45 | 12230.6 ± 2805.29 | 3716.7 ± 1314.04 | 340024.1 ± 65390.33 | 110230.3 ± 15497.68 | 201145.6 ± 21406.63 |
|  | Control at day 5 | 11278.3 ± 5837.94 | 8447.9 ± 3927.0 | 2973.3 ± 1017.85 | 216379.0 ± 62781.11 | 71801.4 ± 17265.47 | 147091.3 ± 46883.13 |
|  | AdipoRon at day 0 | 16437.5 ± 2669.42 | 10213.1 ± 2375.68 | 3716.7 ± 0.0 | 335078.3 ± 58715.16 | 111963.9 ± 12779.64 | 212159.3 ± 42613.52 |
|  | AdipoRon at day 5 | 7198.9 ± 1199.82 | 4034.8 ± 845.83 | 2973.3 ± 1017.85 | 174957.8 ± 18878.67 | 49553.1 ± 7051.68 | 105210.1 ± 6589.37 |
|  | AdipoRonPEG_5_ at day 0 | 19797.0 ± 3523.66 | 11474.0 ± 1631.86 | 4088.3 ± 831.07 | 358570.9 ± 58282.32 | 127711.1 ± 26772.71 | 224332.4 ± 35925.97 |
|  | AdipoRonPEG_5_ at day 5 | 3599.4 ± 848.4 | 4287.0 ± 935.1 | 2230.0 ± 831.07 | 88406.3 ± 22461.25 | 33950.3 ± 10616.3 | 67386.7 ± 20818.51 |

**Table S8**. Average (pg/mg) and standard deviation of ceramides in liver, pancreas, scWAT and serum of HFD-mice with and without (control) treatment of AdipoRon and AdipoRonPEG_5_.

| **Tissue** | **Sample Name** | **Cer 14:0** | **Cer 16:0** | **Cer 18:0** | **Cer 18:1** | **Cer 20:0** | **Cer 22:0** | **Cer 24:0** | **Cer 24:1** | **Cer 26:0** | **Cer 26:1** |
| --- | --- | --- | --- | --- | --- | --- | --- | --- | --- | --- | --- |
| Liver | Control | 8627.2 ± 3075.8 | 8157.2 ± 1954.85 | 26204.5 ± 7499.06 | 0 | 1535518.1 ± 605423.58 | 18051080.3 ± 1922948.87 | 8742255.8 ± 1643612.35 | 6569561.5 ± 951767.15 | 80965.7 ± 15474.64 | 67838.0 ± 8749.71 |
|  | AdipoRon | 11180.9 ± 7129.23 | 15452.5 ± 14214.83 | 47128.6 ± 39264.7 | 0 | 2021924.3 ± 1132557.52 | 21221267.5 ± 1938950.14 | 8844684.8 ± 1440139.38 | 7621686.9 ± 1072827.16 | 108319.5 ± 12837.73 | 87632.7 ± 14143.63 |
|  | AdipoRonPEG_5_ | 10700.6 ± 4986.87 | 10811.2 ± 3038.11 | 41588.7 ± 18827.94 | 0 | 1285175.8 ± 314117.31 | 17214621.7 ± 3730792.66 | 13003125.0 ± 3100177.0 | 11982004.5 ± 3750645.21 | 114936.8 ± 43584.75 | 108478.1 ± 38829.61 |
| Pancreas | Control | 28.1 ± 7.15 | 27.8 ± 13.41 | 33.6 ± 13.91 | 0 | 1869.2 ± 485.78 | 7142.0 ± 1363.15 | 21073.9 ± 5015.58 | 10379.1 ± 3084.83 | 163.2 ± 32.27 | 84.4 ± 17.01 |
|  | AdipoRon | 26.4 ± 4.51 | 26.1 ± 10.18 | 28.9 ± 6.11 | 0 | 1529.7 ± 140.3 | 7541.1 ± 1118.82 | 21064.5 ± 2932.0 | 10791.6 ± 1783.65 | 155.6 ± 36.08 | 81.6 ± 16.04 |
|  | AdipoRonPEG_5_ | 20.8 ± 6.06 | 28.6 ± 10.14 | 31.4 ± 12.12 | 0 | 1410.3 ± 372.3 | 5266.0 ± 1349.3 | 16049.4 ± 4089.83 | 7744.9 ± 1489.17 | 162.6 ± 42.67 | 88.6 ± 17.21 |
| scWAT | Control | 32399.0 ± 9102.06 | 218505.1 ± 50885.9 | 83594.8 ± 34656.8 | 17778 ± 3428 | 193441.1 ± 44359.16 | 834503.3 ± 44224.29 | 2356874.3 ± 1005257.23 | 2757893.1 ± 898990.75 | 26538.8 ± 10202.91 | 26240.0 ± 9277.77 |
|  | AdipoRon | 41670.5 ± 9888.98 | 200569.4 ± 60832.18 | 90968.7 ± 40221.97 | 24648 ± 8680 | 189778.3 ± 38166.38 | 835762.6 ± 112294.65 | 2316061.3 ± 1012651.99 | 2616280.7 ± 714833.34 | 25284.4 ± 8047.31 | 27852.3 ± 8212.8 |
|  | AdipoRonPEG_5_ | 32099.4 ± 10520.94 | 191093.1 ± 51596.09 | 82819.6 ± 17887.79 | 21443 ± 6799 | 147177.5 ± 8589.73 | 712843.5 ± 129405.16 | 2685762.3 ± 976754.88 | 3013765.2 ± 650894.91 | 24885.1 ± 6744.06 | 30570.9 ± 6986.86 |
| Serum | Control at day 0 | 9165.8 ± 1597.64 | 27970.9 ± 6484.08 | 14211.7 ± 4278.87 | 0 | 197210.3 ± 99402.3 | 2437645.8 ± 789982.85 | 1348478.8 ± 349772.03 | 1115446.9 ± 317216.67 | 10329.0 ± 3128.84 | 0 |
|  | Control at day 5 | 7638.2 ± 2338.7 | 23189.5 ± 9068.02 | 8929.5 ± 2369.64 | 0 | 146917.7 ± 41131.78 | 1499579.3 ± 375101.87 | 1198808.4 ± 370005.19 | 668548.1 ± 101026.53 | 10329.0 ± 3915.48 | 0 |
|  | AdipoRon at day 0 | 8402.0 ± 1707.95 | 25341.1 ± 4464.54 | 10061.4 ± 1406.12 | 0 | 144673.7 ± 32033.84 | 2303972.0 ± 309633.22 | 1443539.8 ± 157739.65 | 986691.7 ± 101968.89 | 10057.2 ± 2818.25 | 0 |
|  | AdipoRon at day 5 | 9165.8 ± 853.97 | 17212.8 ± 1488.18 | 8049.1 ± 1033.29 | 0 | 135037.6 ± 17909.21 | 1736100.3 ± 185474.31 | 1412623.3 ± 289722.52 | 771667.5 ± 65564.62 | 11959.9 ± 2614.25 | 0 |
|  | AdipoRonPEG_5_ at day 0 | 9547.7 ± 2338.7 | 29166.2 ± 4917.61 | 8552.2 ± 1447.69 | 0 | 161041.9 ± 19909.93 | 2248954.3 ± 477468.89 | 1497571.4 ± 240898.06 | 1109542.1 ± 131449.16 | 11416.3 ± 5402.24 | 0 |
|  | AdipoRonPEG_5_ at day 5 | 9929.6 ± 3137.7 | 15778.4 ± 1918.13 | 13708.7 ± 1433.97 | 0 | 132529.6 ± 32152.61 | 1388023.2 ± 196403.0 | 1824217.1 ± 192203.29 | 1024425.3 ± 215659.12 | 16852.6 ± 6836.06 | 0 |
